# Impacts of California Proposition 47 on crime in Santa Monica, California

**DOI:** 10.1371/journal.pone.0251199

**Published:** 2021-05-19

**Authors:** Jennifer Crodelle, Celeste Vallejo, Markus Schmidtchen, Chad M. Topaz, Maria R. D’Orsogna

**Affiliations:** 1 Department of Mathematics, Middlebury College, Middlebury, VT, United States of America; 2 Mathematical Biosciences Institute, The Ohio State University, Columbus, OH, United States of America; 3 Laboratoire Jacques-Louis Lions, Sorbonne Université, Paris, France; 4 Institute for the Quantitative Study of Inclusion, Diversity, and Equity, Williamstown, MA, United States of America; 5 Department of Mathematics and Statistics, Williams College, Williamstown, MA, United States of America; 6 Department of Computational Medicine, UCLA, Los Angeles, CA, United States of America; 7 Department of Mathematics, CSUN, Los Angeles, CA, United States of America; Universidade Estadual de Maringa, BRAZIL

## Abstract

We examine patterns of reported crime in Santa Monica, California before and after the passage of Proposition 47, a 2014 initiative that reclassified some non-violent felonies as misdemeanors. We also investigate impacts of the opening of four new light rail stations in 2016 and of increased community-based policing starting in late 2018. Our statistical analyses of reclassified crimes—larceny, fraud, possession of narcotics, forgery, receiving/possessing stolen property—and non-reclassified ones are based on publicly available reported crime data from 2006 to 2019. These analyses examine reported crime at various levels: city-wide, within eight neighborhoods, and within a 450-meter radius of the new transit stations. Monthly reported reclassified crimes increased city-wide by approximately 15% after enactment of Proposition 47, with a significant drop observed in late 2018. Downtown exhibited the largest overall surge. Reported non-reclassified crimes fell overall by approximately 9%. Areas surrounding two new train stations, including Downtown, saw significant increases in reported crime after train service began. While reported reclassified crimes increased after passage of Proposition 47, non-reclassified crimes, for the most part, decreased or stayed constant, suggesting that Proposition 47 may have impacted reported crime in Santa Monica. Reported crimes decreased in late 2018 concurrent with the adoption of new community-based policing measures. Follow-up studies needed to confirm long-term trends may be challenging due to the COVID-19 pandemic that drastically changed societal conditions. While our research detects changes in reported crime, it does not provide causative explanations. Our work, along with other considerations relevant to public utility, respect for human rights, and existence of socioeconomic disparities, may be useful to law enforcement and policymakers to assess the overall effect of Proposition 47.

## Introduction

On November 4, 2014, voters of the state of California passed Proposition 47 (hereafter Prop. 47), also known as the “Criminal Sentences. Misdemeanor Penalties. Initiative Statute.” or “The Safe Neighborhoods and Schools” Act. The referendum, which was approved with 59.6% of the vote, went into effect the following day, November 5, 2014 [1]. Prop. 47 imparted three broad changes to felony sentencing laws in the state of California: (i) certain non-violent theft and drug possession offenses would be reclassified from felonies to misdemeanors; (ii) those serving sentences for the reclassified offenses would be allowed to petition courts for re-sentencing; and (iii) those who had completed felony sentences now classified as misdemeanors would be able to petition courts to amend their criminal records. Felonies reclassified as misdemeanors under Prop. 47 include shoplifting, attempted shoplifting, grand theft auto, receiving stolen property, forgery, fraud, and writing bad checks—each up to a maximum monetary value of 950 USD. Possession of most illegal drugs for personal use, including methamphetamine, heroin, and cocaine, was also reclassified as a misdemeanor. The law allows for some exceptions. For instance, reclassification may not apply if perpetrators have a criminal record including violence or sexual offenses.

Prop. 47 was part of a series of initiatives designed to lessen California’s incarcerated population in response to allegations of inadequate inmate medical and mental health care, amounting to cruel and unusual punishment. In 2009, federal courts required the state to reduce prison overcrowding and set an occupancy threshold of 137.5% of design capacity to guarantee inmates’ Eighth Amendment rights. On May 23, 2011, and upon appeal by the State of California, the US Supreme Court upheld this decision in *Brown vs. Plata*. California’s prison population would have to decrease from approximately 156,000 to 110,000 individuals [2]. To comply with federal orders, state lawmakers enacted significant legislative reforms over the years, including Prop. 47. More specifically, Assembly Bill (AB) 109, also known as the Public Safety Realignment Bill, and Assembly Bill 117, also known as the Criminal Justice Realignment Bill, were approved and went into effect on October 1, 2011 [3]. These laws allow individuals convicted of certain non-violent crimes to serve their sentences in county facilities, under house arrest, or in alternative sentencing schemes, rather than in state prisons. Overall, 500 criminal statutes were amended and penalties for parole violations were reduced. On November 6, 2012, voters also approved Prop. 36, which revised California’s 1994 Three Strikes Law mandating a sentence of 25 years to life for those convicted of a third felony. Under Prop. 36, to be considered a strike, the third offense must be a serious or violent felony, or the perpetrator must have been previously convicted of murder, rape, or child molestation.

Although the state prison population fell after enactment of AB 109 and AB 117, it was only after passage of Prop. 47 that the incarcerated population dropped below the 2009 court-mandated target [4–6]. One study found a 50% decline in the number of individuals being held or serving sentences for the reclassified crimes [7]. Prop. 47 also stipulated that any resulting monetary savings should be diverted to crime prevention programs targeting youth and recidivists. The Safe Neighborhoods and Schools Fund was specifically created to manage these savings, estimated to be between 150 and 250 million USD per year. To date, 65% of payments have been distributed to the Board of State and Community Correction, with the Department of Education and the Victim Compensation and Government Claims Board receiving minor percentages [8]. Reducing penalties for drug possession may have also lessened racial and ethnic disparities in the California criminal justice system [9].

While Prop. 47 helped reduce incarceration, determining its effects on the reclassified crime rates has proven more controversial. Several parties, including law enforcement officials, district attorneys, and mayors, point to the law for rising crime [10–13]. Some studies link moderate [14] or sustained [15, 16] crime increases to the enactment of Prop. 47, while other groups maintain that current data is inconclusive and that a longer term perspective is necessary [17]. Aside from the disputed effects of Prop. 47 on crime rates, it has also been claimed that the new law brought unintended consequences such as the elimination of DNA collection for the reclassified crimes, restrictions in arresting repeat offenders, declines in the reporting of crimes as victims learned that police would not be able to apprehend and punish perpetrators, and lessened likelihood of habitual drug users seeking treatment [18]. A preliminary analysis conducted state-wide by the California Police Chiefs Association found that the consequences of Prop. 47 are not homogeneous among cities of comparable size and that county specific factors, such as efficacy of monitoring and treatment programs, and how probation and/or incarceration are handled locally, may affect crime rates [10]. Judging the outcomes of Prop. 47 has led to a contentious debate within academic, political, and community settings, culminating in a growing movement to reverse some of its reforms through a 2020 ballot initiative that was eventually rejected at the ballot box. Public safety agencies, caught between opposite viewpoints on the overall positive or negative societal effects of Prop. 47, have often expressed the need to better understand its consequences to optimize operations and budgets, to improve procedures, and to share impartial findings with stakeholder groups [19].

Our research investigates the impacts of Prop. 47 on crime rates in the coastal city of Santa Monica, California, population 91,411 (2019). Located in Los Angeles County, the city is bordered by Los Angeles proper and the Pacific Ocean. Its downtown core has recently undergone intense revitalization fueled by high-tech start-ups, increased tourism, and the 2016 opening of the Metro Expo Line light rail extension. The latter now connects the beach with nearby Culver City and inner Los Angeles neighborhoods through seven new stations, of which four are located within municipal borders. The city has also experienced rising housing costs, the displacement of long-term tenants, and increasing levels of homelessness [20, 21]. In recent years both the Santa Monica Police Department (SMPD) and the local press have reported increases in crime, including robbery, burglary, aggravated assault, and homicide, with large numbers of repeat offenders [22–25]. Dedicated social media accounts and resident neighborhood groups [26–29] have been awash with images, anecdotal evidence, and speculation on the root cause of these trends. Passage of Prop. 47 is among the theories offered to explain the rise in crime; another is the opening of the Expo Line allowing for easier transportation to and from the city [30–32].

After a change in leadership in May 2018, the SMPD launched a series of new public safety initiatives. These included hiring twenty new police officers, increasing patrolling and outreach efforts, establishing a dedicated unit to analyze crime trends, deploying nightly security guards, adding lighting and CCTV cameras to public garages, and even limiting the hours of operations of some businesses that attracted large amounts of crime [33]. The SMPD also increased its engagement with people experiencing homelessness and former inmates, helping them connect to services. Among the newly established programs are the Neighborhood Resource Officers to facilitate community-oriented policing, the Homeless Liaison Program, to assist the unhoused, and the Downtown Business Services Unit to improve communication with business owners. These initiatives are reported to have mitigated crime in the city, especially those affecting quality of life [34, 35].

As part of its pledge towards greater transparency, the SMPD maintains a publicly available crime database that provides dates, types, and locations of crimes reported within its jurisdiction starting from January 2006 until the present day [36]. Motivated by the many changes to the city, and to better quantify how reported crime has changed over the past thirteen years, we perform statistical analyses on this large body of data with a specific focus on identifying possible effects of Prop. 47. Our data analysis is meant as a first step in quantifying long-term crime trends in Santa Monica, and as a way to go beyond casual information and/or personal opinion. Throughout our work, in every instance where we discuss changes to crime trends, it is important to note that any increase or decrease we present applies only to the reported crimes listed by the SMPD. This qualifier is crucial, as the SMPD data may not be an unbiased representative sample of the actual crimes committed. For example, the data may be affected by biases in collection methods, changes to police routines, changes in the public’s habits, and more. We caution especially strongly that our results must not be used as justification for altering patterns of policing.

With the caveats above in mind, we find that the average monthly number of reported reclassified crimes increased overall by about 14.7% after enactment of Prop. 47. The sharp increase emerges in the latter part of 2014, concurrent with passage and implementation of the new law. We observe a decrease in reported reclassified crimes towards the end of 2018. This decrease persists through 2019 and is concurrent with the new police initiatives mentioned previously. Overall, the average monthly number of non-reclassified crimes fell by 9.2%. Longer term studies would be needed to determine whether this decrease will stabilize in the future. A geographical analysis reveals that reported reclassified crimes after passage of Prop. 47 increased or stayed constant in all but one of the eight Santa Monica neighborhoods, with significant monthly rises in the Downtown, North of Montana, and Ocean Park neighborhoods (+37.2%, +13.2%, and +12.0%, respectively). In contrast, reported non-reclassified crimes appear to have decreased in all districts, except for Downtown, which saw a 14.4% increase.

Finally, we analyze monthly average reported crime rates within 450 meters of the four new Expo Line train stations opened in Santa Monica in May 2016. Of these, the Downtown Santa Monica, Expo/Bundy, and 17^th^ Street/Santa Monica College stops exhibit a statistically significant increase for all reported crimes after May 2016; the difference is not significant for the 26^th^ Street/Bergamot station. Increases are similar for both reclassified (+30.6%) and non-reclassified (+33.5%) crimes at Downtown Santa Monica. At 17^th^ Street/Santa Monica College, in contrast, the percent increase of reclassified crimes (+38.6%) is much larger than that of non-reclassified crimes (+23.1%). Finally, reclassified crimes increase at 26^th^ Street/Bergamot (+30.0%) but non-reclassified ones do not vary appreciably. These results may suggest that Prop. 47 led to differential reported crime increases at the Expo Line train stations.

Our study uses several methodological approaches. We use a Welch’s t-test to show that the average monthly number of crimes affected by Prop. 47 after November, 2014 is significantly larger than prior to that date. We further decompose the full 2006–2019 crime time series into three components: trend, periodic seasonality, and random residual. The resulting trend increases around the end of 2014, as determined by change-point analysis and segmented regression. Spatial data plays a crucial role in our analysis of crime trends in each of the eight neighborhoods that comprise the city of Santa Monica, and the more localized areas associated with the opening of the Expo Line.

## Data

We use data from an open source file managed and updated by the Santa Monica Police Department (SMPD) from January 2006 to the present day [36]. The raw data includes records of crimes known to the city (as reported by the public and/or known through officer-initiated activity), the Uniform Crime Reporting (UCR) classification code as determined by the FBI [37], a description of the type of crime, the date on which it occurred, and the latitude and longitude of its location. To better manage the information and perform statistical analyses on categories with a sufficient amount of data, we collapse some original crime categories into coarser ones. For instance, we group “aggravated assault with a firearm,” “general aggravated assault,” “aggravated assault with hands,” “aggravated assault with knife,” and “aggravated assault with other weapon,” under the new general category “assault.” We also set a threshold of 950 counts per category (close to the crime count for disorderly conduct) for the non-reclassified crimes, so that if this minimum number is not met over the 2006–2019 period, the category is excluded from our analysis due to insufficient data. The first excluded (that is, next most populated) category is the unlawful carrying and/or possession of a weapon, for which a total of 474 crimes are tallied, just over half the imposed threshold. Other crimes that do not meet the threshold are arson, embezzlement, blackmail and homicide. Misappropriation of property appears in the database only from 2010 onwards so we also discard this category from our analysis. Finally, we discard all incomplete or corrupted entries. The total crime count for crimes not included in our analysis represents 2% of all crimes in the database.

**Fig 1:**
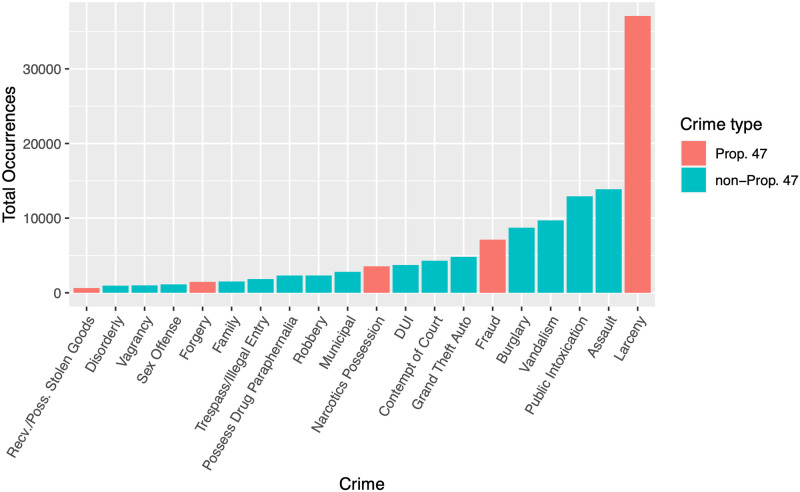
Reported crimes in Santa Monica, CA, 2006–2019, by crime type. Reclassified crimes are as follows: receiving/possessing stolen goods, forgery, narcotics possession, fraud, and larceny. All others do not fall under the provisions of Prop. 47. Data is from Table 1.

**Fig 2:**
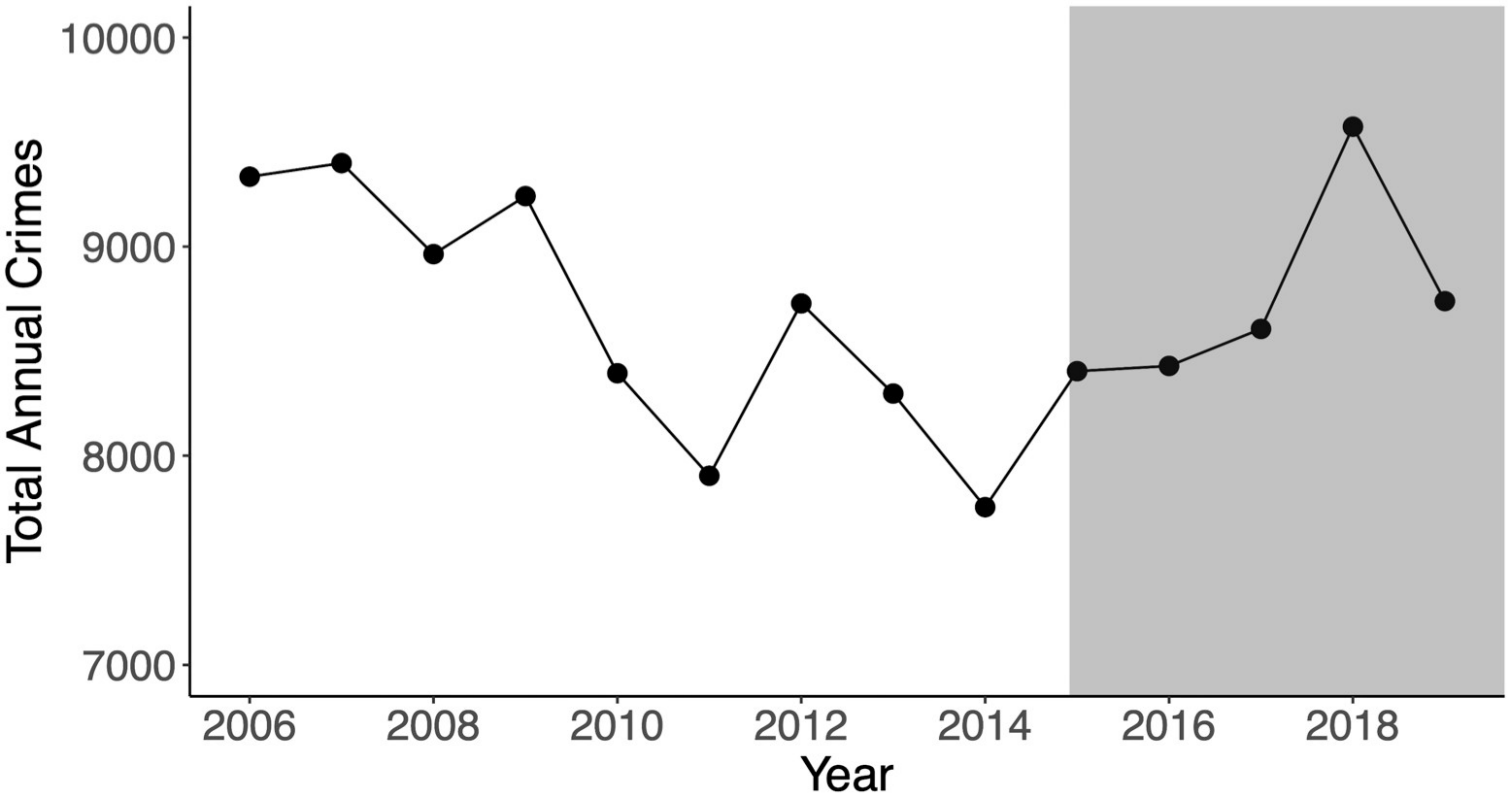
Reported crimes in Santa Monica, CA, 2006–2019, by year. The gray area indicates the years during which Prop. 47 has been enforced. Prop. 47 went into effect during November, 2014, the calendar year which had the fewest reported crimes. The Santa Monica Police Department launched new community policing initiatives in September, 2018. We note a sharp decline in reported crime for 2019.

Table 1 lists the broad crime categories that were reclassified from felonies to misdemeanors under Prop. 47 and their respective 2006–2019 city-wide counts. These categories are larceny, fraud, narcotics possession, forgery, and receiving/possessing stolen property. We refer to these collectively as “Prop. 47 crimes” or “reclassified crimes.” Crimes not affected by Prop. 47 are also listed in Table 1 as “non-Prop. 47 crimes.” We will also refer to them as “non-reclassified crimes.” The crime database is updated by the Santa Monica Police Department daily. For most of our analyses we consider monthly, or in some cases yearly, aggregates. Later, we will compare temporal trends between reclassified and non-reclassified crimes to examine possible effects of the 2014 initiative. Fig 1 gives an overall view of the data. Larceny, one of the Prop. 47 offenses, has the highest overall incidence followed by assault and public intoxication, both non-Prop. 47 crimes. Fig 2 displays total (reclassified and non-reclassified) reported crimes by year, from 2006 through 2019.

**Table 1:** Reported crimes in Santa Monica, CA, 2006–2019, by crime type.

**Prop. 47 Crimes**	**Count**
Larceny	37,082
Fraud	7,121
Narcotics possession	3,549
Forgery	1,458
Receiving/possessing stolen property	636
Total	49,846
**non-Prop. 47 Crimes**	**Count**
Assault	13,870
Public intoxication	12,926
Vandalism	9,699
Burglary	8,724
Grand theft auto (GTA)	4,812
Contempt of court	4,295
DUI	3,724
Municipal crimes	2,804
Robbery	2,317
Possession of drug paraphernalia	2,315
Trespass/illegal entry	1,844
Family crimes	1,510
Sex offenses	1,128
Vagrancy	995
Disorderly conduct	957
Total	71,920
All Crimes	121,766

The Prop. 47 crimes are those that were reclassified in 2014, whereas the non-Prop. 47 crimes are those that were not. See Data for explanation of what crimes we include.

Prop. 47 imposed a maximum monetary value of 950 USD for crimes to be reclassified as misdemeanors; however, no dollar amount information is specified in the data set we examined. We used ancillary information to determine which categories should fall under the Prop. 47 header, depending on their typical economic value. For example, a closer inspection of the database reveals that larceny in Santa Monica is mostly the unlawful taking of objects or parts from motor vehicles, of objects from buildings, of used bicycles or shoplifting, the value of which we estimate to be below the 950 USD threshold. Less frequent larceny crimes are shoplifting, pickpocketing, purse snatching and tampering with coin machines, which we also assume to be below the 950 USD threshold. Since the average street value of cocaine, heroin or methamphetamine doses for personal use is well below the 950 USD threshold, we also include possession of narcotics in the Prop. 47 reclassified list. The Federal Reserve estimates that for the year 2015, fraud from bad checks, general-purpose transactions, and credit card accounts resulted in 62 million single payments for a total of 8.3 billion USD, averaging 135 USD per transaction [38]. We thus include fraud and forgery in the Prop. 47 crime list. Finally, we do not include grand theft auto in the list of Prop. 47 crimes, since as per conversations with the SMPD, the typical value of stolen vehicles exceeds 950 USD, and thus this crime may fall outside the scope of Prop. 47. The SMPD also confirmed that the monetary value associated with all the Prop. 47 crimes listed in Table 1 is usually under the 950 USD threshold imposed for reclassification purposes. We do not adjust crime counts for population change since the number of Santa Monica inhabitants has remained fairly stable in the thirteen year period under investigation. The city tallied approximately 87,000 residents in 2006, and after peaking at 93,000 in 2015, the population is currently estimated to be 91,411 [39].

## Results: City-wide

**Fig 3:**
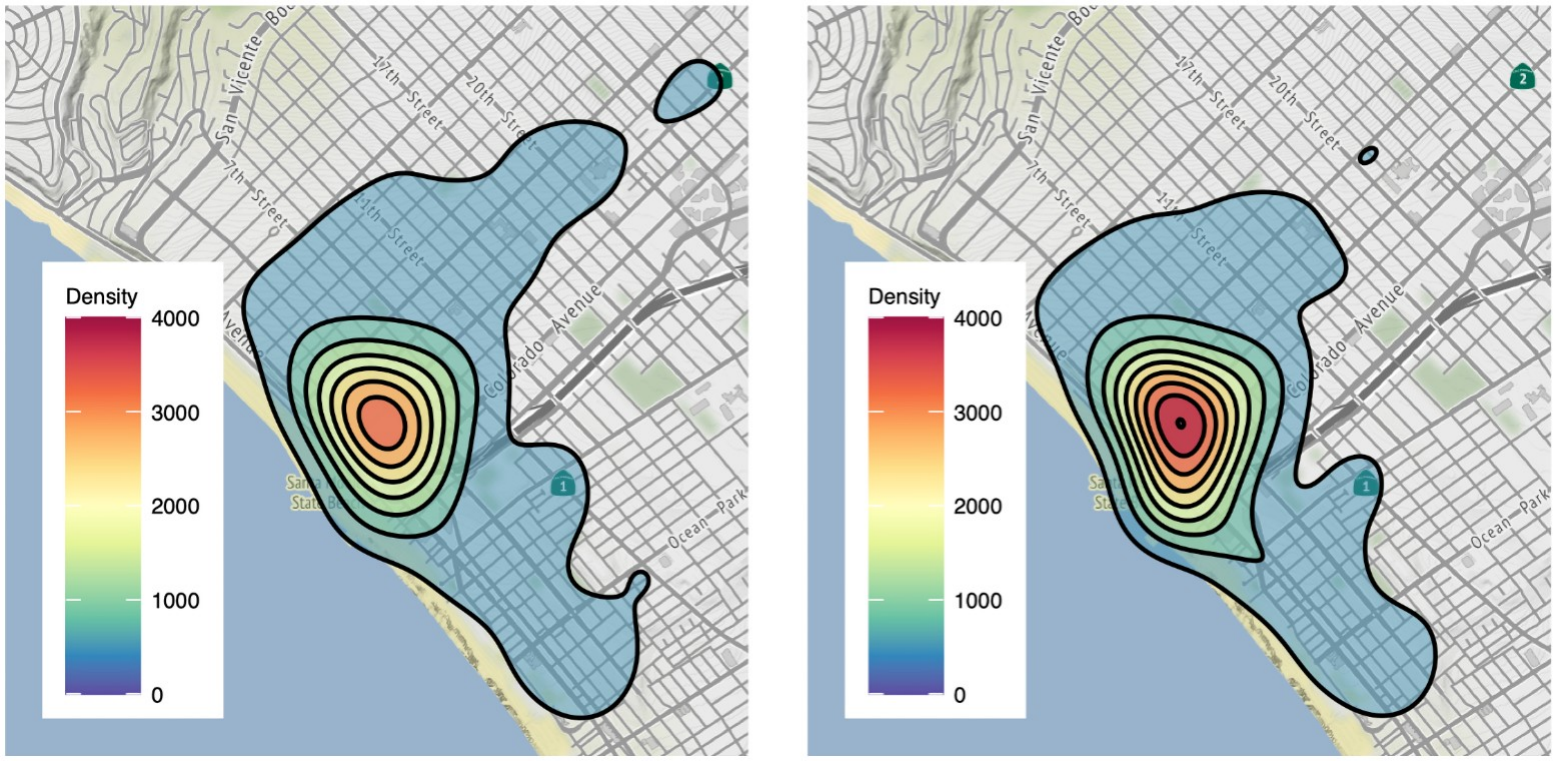
Density of average annual reported larceny incidents in Santa Monica, CA, before Prop. 47 (left) and after Prop. 47 (right). The right-hand panel displays more intense coloring in the central contours, reflecting higher incidence of larceny after implementation of Prop. 47. Though these maps are restricted to larceny, all crime types are more prevalent Downtown compared to residential areas. Created using OpenStreetMaps.

As motivation, we plot in Fig 3 a density map of the average annual reported incidence of larceny, a reclassified crime, before and after implementation of Prop. 47. Most events are located in downtown Santa Monica, with the average annual reported crime density increasing after 2014, as can be seen by the more intense coloring in the right-hand panel. To better understand any associations between Prop. 47 and reported crime city-wide, we now perform a series of statistical analyses: a Welch’s t-test on average monthly reported crimes before and after Prop. 47; a seasonal-and-trend decomposition to study the time series of monthly reported crime; change-point detection on the aforementioned trend; and segmented regression. All of these analyses suggest an association between the enactment of Prop. 47 and an increase in reported crime.

### Average monthly reported crime

**Fig 4:**
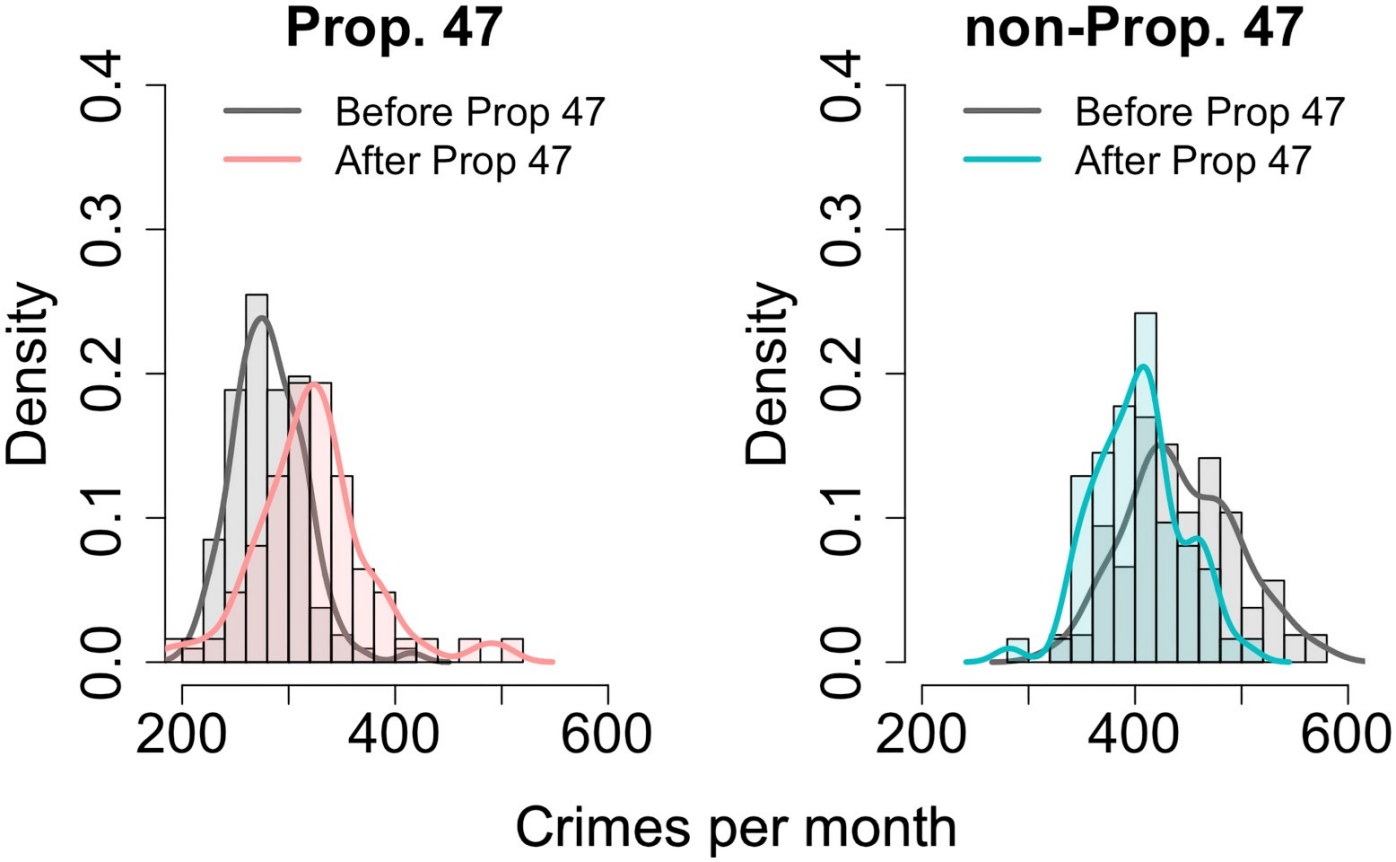
Reported crimes per month in Santa Monica, CA, before and after implementation of Prop. 47 in November, 2014. For Prop. 47 crimes (left panel) the monthly average before implementation of the new law was 281.4 crimes, whereas after November, 2014 the average was 322.9 crimes per month. A Welch’s t-test shows that this 14.7% increase is statistically significant (*p* < 0.05). The same computations applied to non-Prop. 47 crimes (right panel) show that, to the contrary, non-reclassified crimes decrease by 9.2%, from 443.2 to 402.4 per month, after passage of the initiative. A Welch’s t-test shows that this 9.2% decrease is statistically significant (*p* < 0.05).

To quantify the effects of Prop. 47 on reported crime rates in Santa Monica, we compute the average number of monthly offenses subject to reclassification before and after the law’s passage. For comparison, we perform the same analysis on reported non-Prop. 47 crimes. Although Prop. 47 took effect on November 5, 2014, we group all November, 2014 events as occurring after passage of the new law since we bin data by month. Fig 4 displays before-and-after histograms for sets of crimes. The Prop. 47 crime distribution shifts right after November, 2014. On the other hand, the non-Prop. 47 crime distribution shifts to the left. These histograms suggest that after passage of Prop. 47, reported Prop. 47 crimes increased whereas reported non-Prop. 47 crimes decreased.

To determine whether these shifts are statistically significant we use Welch’s unequal variances t-test (Welch’s t-test) to compare the before and after mean monthly count of reported reclassified crimes. This is a two-sample test typically employed to compare two mean values when the respective samples have unequal size or variance [40]. In our specific case, data is available over eight years (106 months) before November, 2014 and only over five years (62 months) after the same date, leading to very different sample sizes and variances. If the occurrence of Prop. 47 crimes listed in Table 1 were not affected by the reclassification process, we would expect the difference between crime counts before and after passage of the law as determined by Welch’s t-test to be negligible.

We denote by *μ*_b_ and *μ*_a_ the mean monthly number of Prop. 47 crimes before and after November, 2014, respectively; *σ*_b_ and *σ*_a_ represent the associated standard deviations, and *N*_b_ and *N*_a_ the respective number of months over which these averages were calculated. The null hypothesis is formulated as there being no difference in the mean values, *μ*_b_ = *μ*_a_, while the alternative hypothesis posits that Prop. 47 led to an increase in the reclassified offenses, *μ*_b_ < *μ*_a_. Our data yields {*μ*_b_, *σ*_b_, *N*_b_}_p47_ = {281.4, 33.2, 106} and {*μ*_a_, *σ*_a_, *N*_a_}_p47_ = {322.9, 53.9, 62}. The ‘p47’ subscript indicates that these statistical values are evaluated on Prop. 47 offenses. To verify whether the before-to-after crime increase is statistically significant we perform a one-tailed Welch’s t-test by calculating the following *t*-statistic

$$\begin{array}{c} t=\frac{\mu_{b}-\mu_{a}}{\sqrt{\frac{\sigma_{b}^{2}}{N_{\text{b}}}+\frac{\sigma_{a}^{2}}{N_{\text{a}}}}}, \end{array}$$ (1)

yielding *t* = 5.5 for the values listed above. This quantity must be compared to the corresponding *t*-value from the Student’s *t*-distribution [41], once the number of degrees of freedom *ν* and the significance level are specified. We denote this reference *t*-value as *t*_s_. Since the before and after Prop. 47 samples are associated with different data sets, each with their own degrees of freedom, we use the Welch-Satterthwaite equation to derive an effective *ν* [42]

$$\begin{array}{c} \nu =\frac{{\left(\frac{\sigma_{\text{b}}^{2}}{N_{\text{b}}}+\frac{\sigma_{\text{a}}^{2}}{N_{\text{a}}}\right)}^{2}}{\frac{\sigma_{\text{b}}^{4}}{N_{\text{b}}^{2}\left(N_{\text{b}}-1\right)}+\frac{\sigma_{\text{a}}^{4}}{N_{\text{a}}^{2}\left(N_{\text{a}}-1\right)}}, \end{array}$$ (2)

from which we obtain *ν* = 89. Finally, we specify a significance level of 0.05 to find the reference value *t*_s_ = 1.66 from the Student’s *t*-distribution. Since this quantity is much smaller than the *t* = 5.5 statistic found from Eq (1), we reject the null hypothesis in favor of the alternative one: the 14.7% increase in the average monthly number of reclassified crimes after the introduction of Prop. 47 is statistically significant.

We perform a similar analysis for the non-reclassified crimes, using {*μ*_b_, *σ*_b_, *N*_b_}_non p47_ = {443.2, 52.6, 106} and {*μ*_a_, *σ*_a_, *N*_a_}_non p47_ = {402.4, 41.5, 62}, where the subscript ‘non p47’ refers to values being evaluated on non-reclassified crimes before and after passage of Prop. 47. We formulate the same null hypothesis as above, *μ*_b_ = *μ*_a_, with the alternative hypothesis set as there being a decrease in the mean monthly number of crimes after November, 2014, *μ*_b_ > *μ*_a_. The *t*-statistic obtained from Eq (1) and the non-Prop. 47 values is *t* = 5.5; Eq (2) yields *ν* = 149, which results in *t*_s_ = 1.66 at the 0.05 significance level. Since *t*_s_ = 1.66 < *t* = 5.5, we reject the null hypothesis in favor of the alternative one: the 9.2% decrease in the average monthly number of non-reclassified crimes after the introduction of Prop. 47 is statistically significant.

#### Seasonal and trend decomposition

To further identify differences in the temporal evolution of the reclassified and non-reclassified offenses, we analyze the entire 2006–2019 crime time series. Temperature variations, seasonal cycles, and the increased criminal opportunities provided by travel and/or shopping during holiday periods are well-known possible crime influencers [43–45]. Although the climate in the coastal Los Angeles basin is typically mild-to-hot and dry throughout the year, heavy rainfall is concentrated in the months of February and March, potentially affecting crime rates. Similarly, large numbers of tourists visit Santa Monica during the summer. It is thus important to remove seasonal effects from the time series to better understand underlying trends. As mentioned in Data, the raw data lists the date of each crime; for convenience we aggregate all occurrences by month to produce a crime time series *Y*(*t*) where *t* is a discrete variable that labels each month from January 2006 to December 2019. In order to separate the main trend in crime progression from possible periodic perturbations, we use the Seasonal and Trend decomposition using Loess (STL decomposition) method on our data set [46]. Here, the full crime time series *Y*(*t*) is decomposed into a trend *T*(*t*), a seasonality *S*(*t*), and a remainder *R*(*t*), so that *Y*(*t*) = *T*(*t*) + *S*(*t*) + *R*(*t*), where *S*(*t*) is periodic and *R*(*t*) represents any residual fluctuations of *Y*(*t*). We discard the multiplicative option where the time series is expressed as a product of its components, *Y*(*t*) = *S*(*t*)*R*(*t*)*T*(*t*), since we expect seasonality effects to remain relatively stable over the temporal arc of our data. We decompose the data using the ‘stl’ function in the R statistical package [47]. The algorithm requires specification of a parameter *w*_trend_, the time-frame over which the data is smoothed; S1 Appendix provides more information.

**Fig 5:**
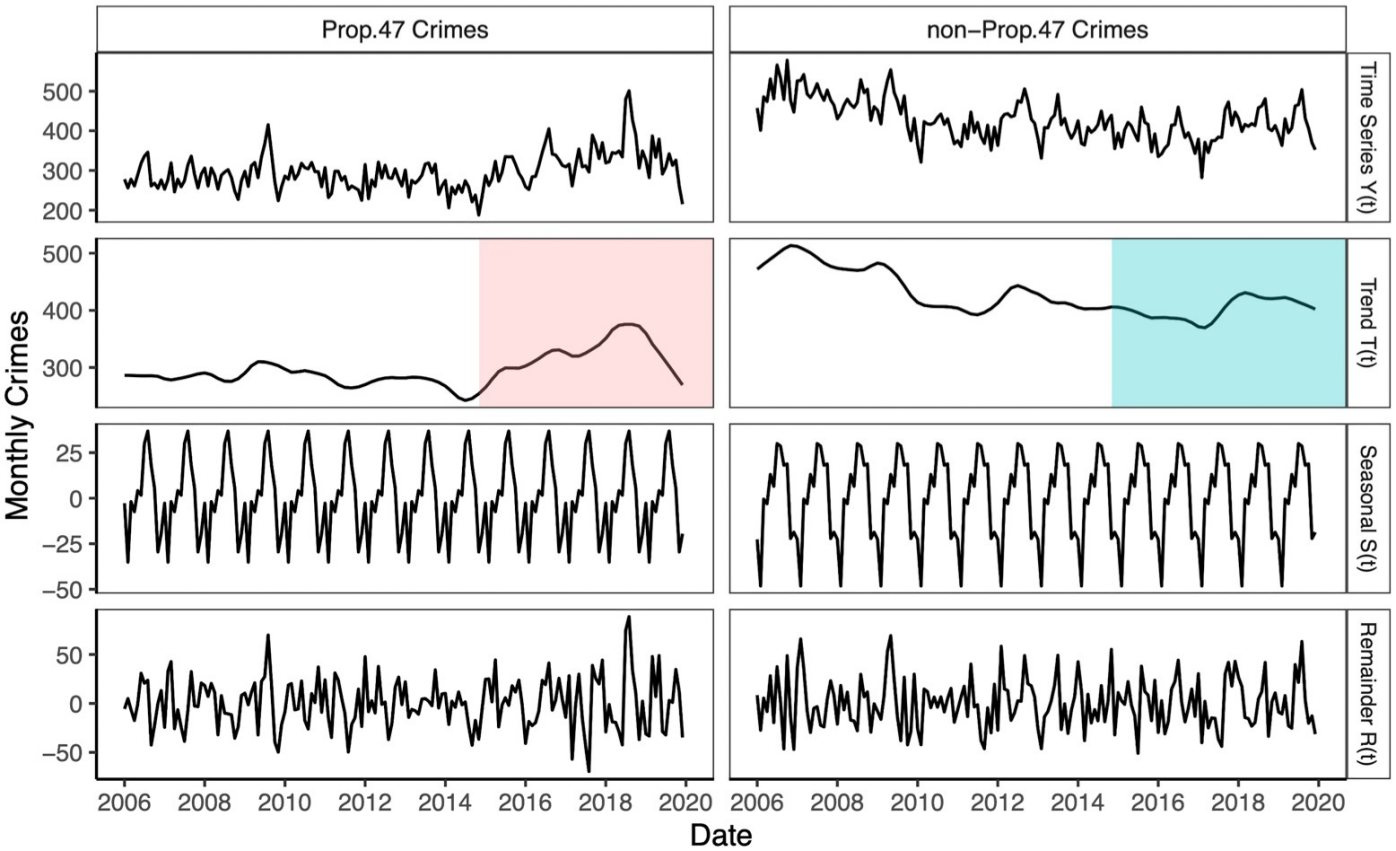
Additive Seasonal and Trend decomposition using Loess (STL) for reported monthly crimes in Santa Monica, CA, 2006–2019. The time series of reported crimes per month *Y*(*t*) is separated into trend *T*(*t*), seasonality *S*(*t*), and remainder *R*(*t*) components. We perform this decomposition for Prop. 47 crimes (left) and non-Prop. 47 crimes (right). The colored regions in the plot of the trend *T*(*t*) indicate the time period following the enactment of Prop. 47. An increase in the trend emerges for the Prop. 47 crimes towards the end of 2014, but not for non-Prop. 47 crimes. For these results, the STL smoothing window is *w*_trend_ = 19.

Fig 5 shows STL decomposition results. The trend *T*(*t*) of reported Prop. 47 crimes begins to increase towards the end of 2014, but no corresponding rise is observed for the non-reclassified crimes. Fig 5 also shows that the increase in Prop. 47 crimes is greater than the decrease in non-Prop. 47 crimes. In fact, non-Prop. 47 crimes appear to be declining from 2014 onwards apart from a slight increase around 2018. This result suggests that the rise in reported reclassified crime should not be attributed to a general pattern of increasing crime rates in the city of Santa Monica. Rather it hints that a specific event in late 2014 may be responsible for the rise in reported Prop. 47 crimes, without playing any role in the dynamics of the non-Prop. 47 ones. We identify this event with the implementation of the new law. A significant drop in the trend *T*(*t*) emerges for Prop. 47 crimes towards the end of 2018, persisting throughout 2019, and concurrent with several new initiatives undertaken by the SMPD to improve public safety and implement more community-based operations.

The season component *S*(*t*) also provides some insight. For both Prop. 47 and non-Prop. 47 crimes, the number of reported offenses increases over spring and summer, reaches a peak in August, and then declines through November. Reported crime increases again throughout the end-of-the-year holiday season, in December and January, and declines in February, during the rainy period. Although the main features of the seasonality components *S*(*t*) of the reclassified and non-reclassified crimes in Fig 5 are similar, some differences arise, most notably behaviors in the spring and fall months. These slight discrepancies might be ascribed to some crimes being more affected by seasonal changes than others.

### Change-point analysis

Having isolated the trend component *T*(*t*) from the time series *Y*(*t*), we determine whether any statistically significant changes in *T*(*t*) arise. If so, we also aim to identify the times at which these changes occur, and the associated confidence intervals. To accomplish these goals, we use change-point analysis, a method that has been applied to disciplines from economics to medicine [48–50]. Once a time series is specified, the basic foundation of change-point analysis is to evaluate a statistical quantity on a subsample of the data immediately prior and immediately after each time point. If the difference between the prior and after quantities surpasses a given threshold, the selected time point is the locus of a change-point, given that some consistency requirements are met. This concept can be applied to the mean, variance, or any moment or derived property of the data [51, 52]. Operationally, the detection of a change-point is framed as a hypothesis test, where the null hypothesis is that there are no change-points and the alternative hypothesis is that at least one exists [48, 49].

We are interested in when the trend *T*(*t*) exhibits the largest rate of change. Thus, we create a new time series *M*(*t*) by evaluating a backward difference on each data point. Let *t*_*i*−1_ < *t*_*i*_ be consecutive times and define *M*(*t*_*i*_) = [*T*(*t*_*i*_) − *T*(*t*_*i*−1_)]/(*t*_*i*_ − *t*_*i*−1_). Because our data points are monthly values, *t*_*i*_ − *t*_*i*−1_ = 1 month and therefore *M*(*t*_*i*_) = *T*(*t*_*i*_) − *T*(*t*_*i*−1_). We compute *M*(*t*) from the trend *T*(*t*) rather than from the original monthly time series *Y*(*t*) because fluctuations in the latter would be amplified when calculating a slope. The trade-off in choosing to work with *T*(*t*) rather than *Y*(*t*) is that the smoothing process in the STL decomposition may affect our analysis. For example the change-points may depend on the smoothing window length *w*_trend_, as discussed in S1 Appendix.

Using the R package ‘mosum’ [53], we perform a change-point analysis on *M*(*t*) to detect where changes to the slope are largest. The algorithm depends on several parameters: the window *G* over which the prior and after subsamples are evaluated; the minimum allowed distance *ηG* between change-points; and the minimum width *ϵG* of a neighborhood of the change-point where the mosum test statistic surpasses the reference threshold for all data points in the *ϵG* neighborhood. These *G*, *η*, *ϵ* parameters affect the location of the change-point and the associated confidence intervals, in addition to *w*_trend_ from the decomposition. See S1 Appendix for more discussion.

**Fig 6:**
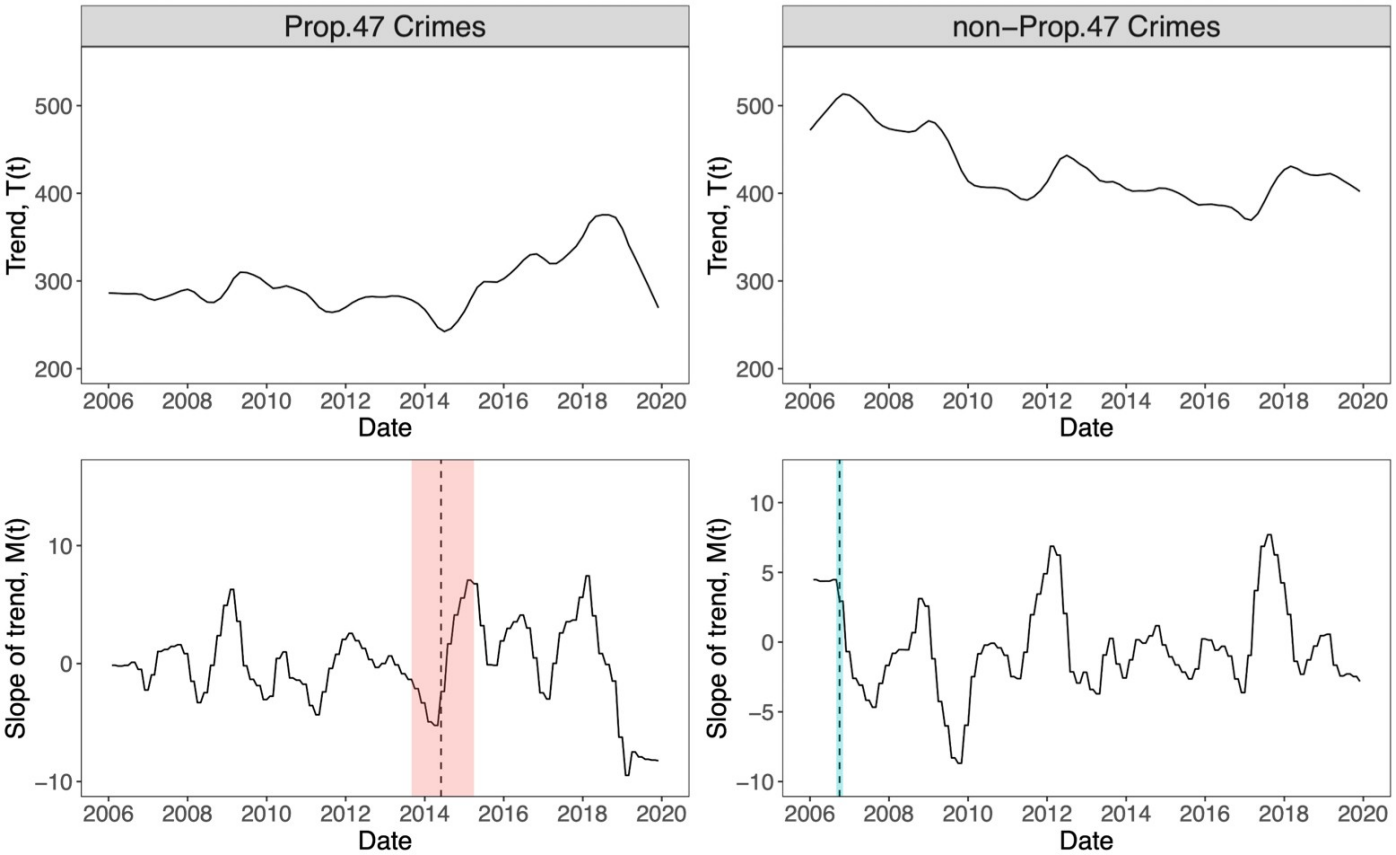
Change-point analysis of reported crime in Santa Monica, CA, 2006–2019. We perform our change-point analysis on the slope *M*(*t*) of the crime trend *T*(*t*) for Prop. 47 crimes (left) and non-Prop. 47 crimes (right). The top panels are the trends *T*(*t*) derived from raw monthly data *Y*(*t*) using STL decomposition with smoothing window *w*_trend_ = 19 months. The lower panels visualize the slopes *M*(*t*), the resulting change-points, and their 95% confidence intervals. Base parameters used in the mosum change-point detection algorithm appear in the main text. The lower left panel for reclassified crimes shows a change-point in June 2014 (dotted line) with a 95% confidence interval between September 2013 and April 2015, which includes November, 2014, when Prop. 47 went into effect. This result is fairly robust to changes in the algorithm’s parameters. For non-reclassified crimes in the right panel, using our base algorithm parameters, the change-point is October 2006 (dotted line) with a 95% confidence interval between September 2006 and November 2006. However, this non-Prop. 47 change-point is strongly dependent on the algorithm parameters. No time-frame emerges that is robust to parameter changes.

Results for Prop. 47 and non-Prop. 47 crimes appear in Fig 6. Using *w*_trend_ = 19 months, *G* = 10 months, *η* = 12, and *ϵ* = 0.5 and detect a change-point for Prop. 47 crimes in June 2014, with a 95% confidence interval that includes November, 2014. Other choices of {*w*_trend_, *G*, *η*, *ϵ*} yield different change-point estimates. Most notably reducing *w*_trend_ will shift the change-point towards later dates. For example *w*_trend_ = 5 months, *G* = 10 months, *η* = 10, *ϵ* = 0.5 yields a change point of August 2014 with a 95% margin of error which also includes November, 2014. We performed change-point analysis for a large set of {*w*_trend_, *G*, *η*, *ϵ*} combinations. For all of them, changes in the rate of Prop. 47 crimes emerge towards the second half of 2014, between June 2014 and August 2014. The choice of *w*_trend_ = 1, which corresponds to building the slope *M*(*t*) from the full time series *Y*(*t*) without any smoothing procedure, typically yields no change-points due to the irregularity of the data, as mentioned above. Some parameter choices allow us to identify additional change-points at the end of 2018. For example, *w*_trend_ = 19 months, *G* = 5 months, *η* = 5, *ϵ* = 0.5 yield October 2018 as a new change-point in addition to June 2014. This is true for other {*w*_trend_, *G*, *η*, *ϵ*} combinations that allow for a smaller window size and a smaller distance between change-points. Thus, while the main change-point remains between June 2014 and August 2014, a minor one also arises towards the end of 2018 for Prop. 47 crimes. The change-point loci for the non-Prop. 47 crimes are more heavily dependent on the chosen {*w*_trend_, *G*, *η*, *ϵ*} parameters, and typically do not extend into 2014. In conclusion, most of the parameter combinations tested yield change-point loci for the Prop. 47 crimes that remain within the June 2014 to August 2014 window, with November, 2014 falling within the 95% confidence interval in all cases.

### Breakpoint analysis

As an alternative to the change-point analysis above, we now perform segmented regression on the reported crime time series *Y*(*t*) and smoothed trend *T*(*t*) for reclassified and non-reclassified crimes. Segmented regression is typically used when abrupt changes are expected in the relationship between an explanatory and a response variable. This relationship is assumed to be piece-wise linear, with segments separated by so-called breakpoints. Under the assumption of a single breakpoint, an initial guess of its location is made and the response variable is fit to two lines, one before and one after the putative breakpoint with the constraint that the overall fit is continuous at the breakpoint itself. The resulting curve is the first estimate on which nonlinear regression models are iterated through least squares (or related) methods until convergence, thereby identifying a breakpoint t*. This procedure can be extended to multiple breakpoints.

**Fig 7:**
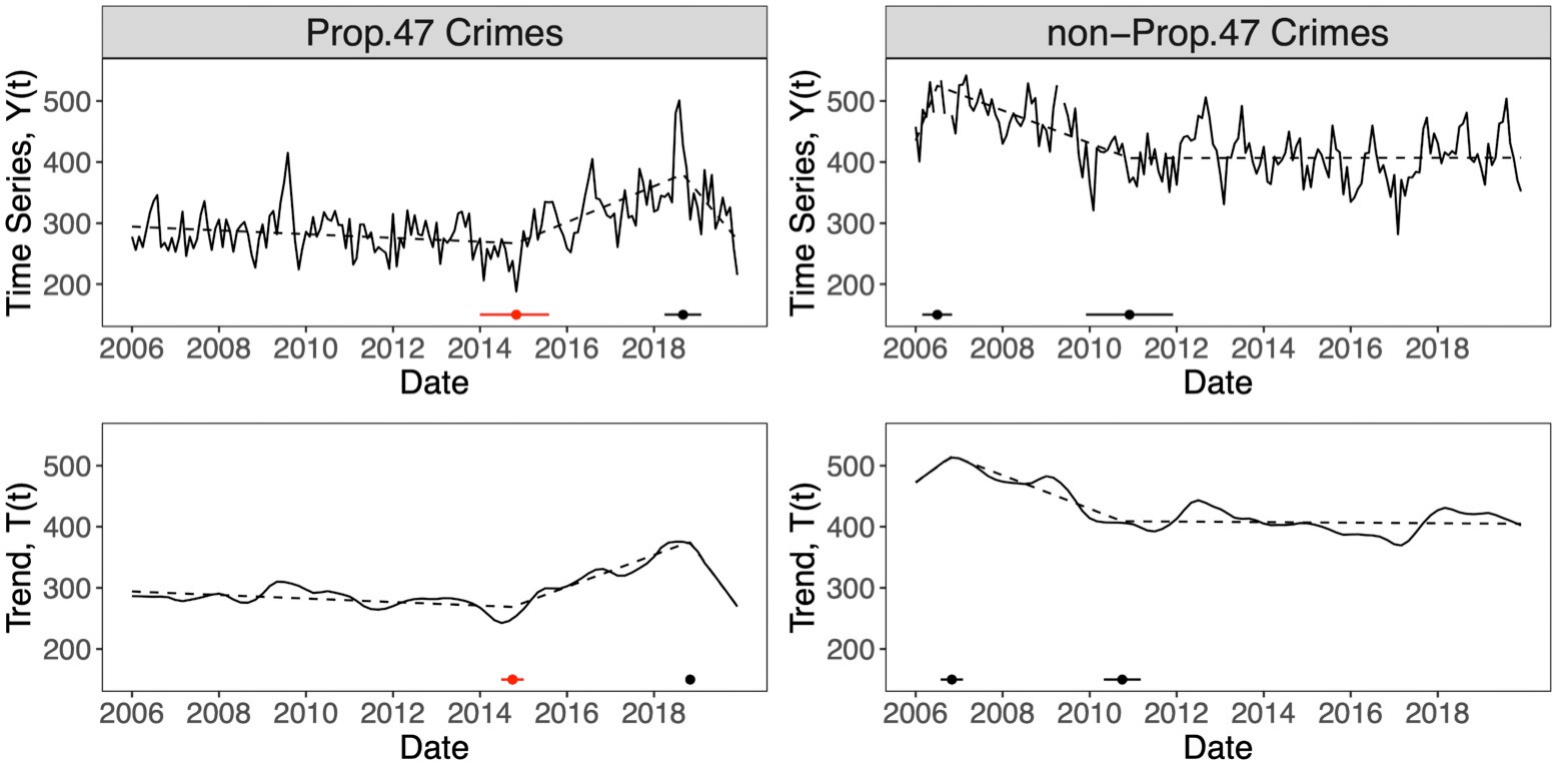
Segmented regression and breakpoint analysis of reported crime in Santa Monica, CA, 2006–2019. According to Davies’ test, the preferred model has two breakpoints. Top and bottom panels show segmented regression for, respectively, the original time series *Y*(*t*) and the smoothed trend *T*(*t*) with *w*_trend_ = 19 months. Left panels are for Prop. 47 crimes and right panels are for non-Prop. 47 crimes. Breakpoints appear as dots and the respective 95% confidence intervals are marked by bars. For Prop. 47 crimes, the first breakpoint (red) is November, 2014 for *Y*(*t*) and October, 2014 for *T*(*t*). Values of 1 < *w*_trend_ < 19 months preserve the *T*(*t*) breakpoint. The second breakpoint (black) is September, 2018 for *Y*(*t*), and October, 2018 for *T*(*t*). The first breakpoint signals a transition to higher crime for both *Y*(*t*) and *T*(*t*), the second one a decrease. For the non-Prop. 47 crimes, breakpoint dates are highly unstable and sensitive to changes in *w*_trend_.

We use the R package ‘segmented’ [54] to perform segmented regression on *Y*(*t*) for Prop. 47 crimes, and on the corresponding *T*(*t*) obtained by setting *w*_trend_ = 19 months. We use Davies’ test within the ‘segmented’ package to determine that the best model contains two statistically significant break points [54]. Results appear in Fig 7. For Prop. 47 crimes, November, 2014 emerges as one of the breakpoints for the monthly time series *Y*(*t*). The corresponding trend *T*(*t*) yields October 2014 as a breakpoint with a 95% confidence interval that includes November, 2014. The location of the *T*(*t*) breakpoint is relatively stable: lower values of 1 < *w*_trend_ < 19 months still yield October 2014 as breakpoint. The second breakpoint for *Y*(*t*) is September 2018. For *T*(*t*) and *w*_trend_ = 19 months it is October 2018. Lower values of *w*_trend_ preserve the October 2018 breakpoint. However *w*_trend_ = 3 and 5 months yield September 2018.

Finally, we perform a segmented regression analysis on the non-Prop. 47 crimes by similarly allowing for two breakpoints. As shown in Fig 7, when using the raw time series *Y*(*t*), breakpoints occur in July 2006 and December 2010 with 95% confidence intervals that do not contain November, 2014. Performing a segmented regression on *T*(*t*) obtained by setting *w*_trend_ = 19 months yields one breakpoint at November, 2006 and one at October, 2010 with 95% confidence intervals that do not include November, 2014 in either case. For the non-Prop. 47 crimes, breakpoints for both *Y*(*t*) and *T*(*t*) are highly unstable and highly sensitive to the choice of *w*_trend_. Furthermore, regardless of the value of *w*_trend_ or the number of breakpoints specified, we found no 95% confidence interval that contains November, 2014, or even a proximal time frame, as a likely breakpoint for non-Prop. 47 crimes. Hence, the abrupt change in the reclassified time series observed in November, 2014 should be associated not with an overall increase in crime, but rather with an event that specifically affected this category of crimes. As discussed above, we identify this event with the implementation of Prop. 47 in November, 2014. Similarly, the second breakpoint evaluated on the Prop. 47 time series and occurring in September 2018 may be associated with the new SMPD policing strategies [33–35] which may have had a stronger impact on Prop. 47 crimes than on non-Prop. 47 ones, for which no corresponding breakpoint was observed. This is because reclassified crimes are generally low-level and quality of life offenses, more easily impacted by the community-based initiatives and the increased patrolling efforts undertaken by the SMPD, such as engagement with vulnerable populations, increased illumination, and physical presence.

## Results: Neighborhood effects

**Fig 8:**
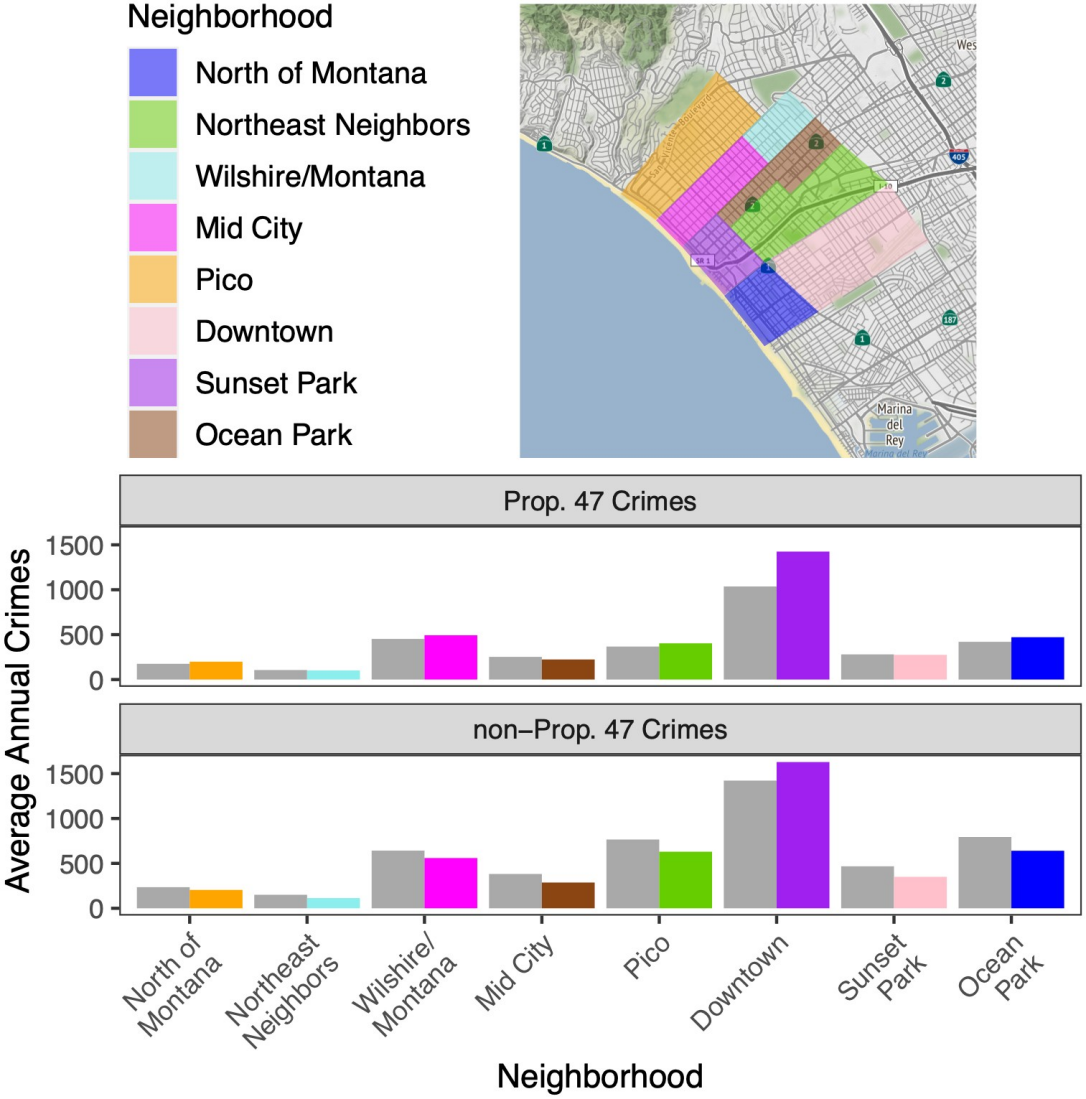
Neighborhoods of Santa Monica, CA, and their average annual reported crimes. (Top) The eight neighborhoods of the city of Santa Monica. To the north of Santa Monica is Pacific Palisades, to the south is Venice, and to the east is West Los Angeles, which are all part of the city of Los Angeles proper. To the west is the Pacific Ocean. Created using OpenStreetMaps. (Bottom): Average annual reported crimes in each neighborhood for Prop. 47 crimes (top) and non-Prop. 47 crimes (bottom). Gray bars indicate the average prior to implementation of the new law in November, 2014. The color-coded bars represent the averages after Prop. 47 came into effect.

The city of Santa Monica is divided into eight neighborhoods marked by specific boundaries as shown in the map in Fig 8. These are: North of Montana, Wilshire/Montana, Northeast Neighbors Association, Mid City, Pico, Downtown, Sunset Park, and Ocean Park. To investigate the geographic effects of Prop. 47, we focus on these districts, plotting for each the average number of reported crimes per year both before and after Prop. 47, and for reclassified and non-reclassified crimes. Gray bars represent the annual reported crimes before November, 2014 and the colored ones those after November, 2014, with the color-coding mirroring that of the neighborhood map. For Prop. 47 crimes, the most impacted areas were the Downtown, North of Montana, and Ocean Park neighborhoods, with 37.2%, 13.2%, and 12.0% increases, respectively, after implementation of Prop. 47. Note that north of Montana has total crime counts that are much lower relative to the Downtown and the Ocean Park neighborhoods. Non-Prop. 47 crimes decreased in all areas, except for Downtown, where crimes increased by an average of 14.4% per year.

**Fig 9:**
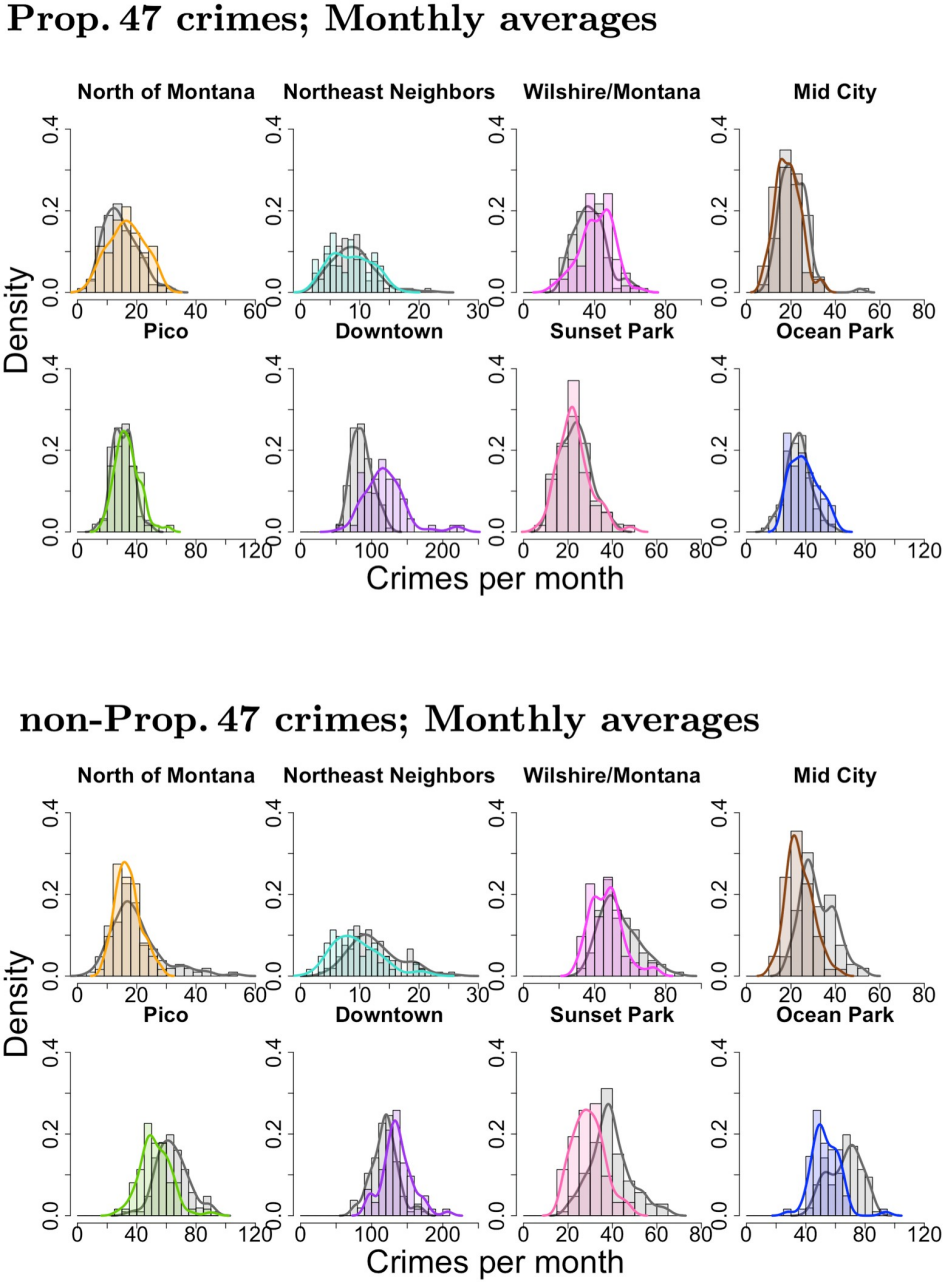
Neighborhood-level analysis of reported crime in Santa Monica, CA, 2006–2019. Normalized histograms and densities of Prop. 47 (top) and non-Prop. 47 (bottom) monthly crimes in the eight Santa Monica neighborhoods. The gray bars and curves represent the time period before November, 2014, and colored ones represent the period after. Table 2 provides summary statistics.

Mirroring the analysis of Results: City-wide, we now construct histograms of average monthly reported crime before and after implementation of Prop. 47 for both reclassified and non-reclassified crimes in each neighborhood; see Fig 9. Gray bars represent reported crimes before November, 2014 and colored bars represent those after. We use the same color scheme as in Fig 8. In all districts, histograms for the Prop. 47 crimes shift to the right or remain unchanged, indicating an increase or stationarity, whereas outcomes for the non-reclassified crimes typically shift to the left, indicating a decrease. The only exception is Downtown, where the non-Prop. 47 crime distribution also shifts to the right.

In Table 2 we quantify whether these shifts are statistically significant by performing Welch’s t-tests. The increases of Prop. 47 monthly crimes in the Downtown, North of Montana, Ocean Park, Pico, Wilshire/Montana neighborhoods are large and statistically significant. On the other hand, we observe statistically-significant decreases in non-reclassified crimes in all neighborhoods except Downtown, where we see a modest increase in reported crime per month. Overall, the largest changes occur Downtown, where the average monthly incidence of reclassified crimes increased by 37.2% after implementation of the new law. This results seems consistent with the fact that reclassified crimes are crimes of opportunity. That is to say, Downtown Santa Monica is rich in crime generators such as shopping, entertainment, and dining venues that attract large numbers of residents and tourists, and may correspondingly draw potential offenders [55]. In any case, our analysis reveals that while reported reclassified crimes increased in most neighborhoods, the strongest effects were felt in areas that were already primed for a criminal surge.

**Table 2:** Statistical tests on reported crime in neighborhoods of Santa Monica, CA, 2006–2019, before and after the passage of Prop. 47.

**Prop. 47 crimes; Monthly averages**				
**Neighborhood**	**Before {*μ*_b_, *σ*_b_}**	**After {*μ*_a_, *σ*_a_}**	***t***	**Significant?**
North of Montana	{14.64, 5.71}	{16.57, 6.02}	2.04	yes, +13.2%
Wilshire/Montana	{37.75, 9.16}	{41.07, 9.63}	2.20	yes, +8.8%
Northeast Neighbors	{8.84, 3.55}	{8.50, 3.44}	0.62	no
Mid City	{21.08, 6.03}	{18.62, 5.59}	2.67	yes, -11.7%
Pico	{30.64, 6.92}	{33.61, 8.37}	2.36	yes, +9.7%
Downtown	{86.48, 14.59}	{118.69, 29.31}	8.09	yes, +37.2%
Sunset Park	{23.37, 6.92}	{22.88, 7.49}	0.42	no
Ocean Park	{35.07, 8.44}	{39.27, 9.32}	2.92	yes, +12.0%
**non-Prop. 47 crimes; Monthly averages**				
**Neighborhood**	**Before {*μ*_b_, *σ*_b_}**	**After {*μ*_a_, *σ*_a_}**	***t***	**Significant?**
North of Montana	{19.61, 8.59}	{17.07, 4.26}	2.56	yes, -13.0%
Wilshire/Montana	{53.60, 10.76}	{46.63, 9.21}	4.44	yes, -13.0%
Northeast Neighbors	{12.57, 4.37}	{9.51, 4.06}	4.57	yes, -24.3%
Mid City	{31.92, 7.51}	{23.94, 5.93}	7.61	yes, -25.0%
Pico	{63.91, 10.98}	{52.61, 10.51}	6.61	yes, -17.7%
Downtown	{118.67, 18.78}	{135.77, 21.4}	5.22	yes, +14.4%
Sunset Park	{39.03, 9.05}	{29.25, 6.97}	7.84	yes, -25.1%
Ocean Park	{66.25, 11.03}	{53.45, 9.63}	7.88	yes, -19.3%

We apply Welch’s t-test to the histogram data visualized in Fig 9 for Prop. 47 (top) and non-Prop. 47 (bottom) crimes. The last column in the table indicates whether changes are statistically significant and shows percent changes to the mean. We highlight significant increases in boldface. The {*μ*_b_, *σ*_b_} quantities are monthly averages and standard deviations of reported crime before implementation of Prop. 47 calculated over *N*_b_ = 106 months. Their post-implementation counterparts are {*μ*_a_, *σ*_a_} calculated over *N*_a_ = 62 months. The Welch’s t-test statistic, *t*, is compared to the Student’s t-distribution reference value *t*_s_ = 1.66. See Results: City-wide for more details.

In S1 Appendix, to analyze time dependent trends at the local level, we perform an STL decomposition in each of the eight Santa Monica neighborhoods, similar to the analysis in Results: City-wide. We find that Downtown is marked by the highest increase in crime after implementation of Prop. 47 but also by the strongest decrease starting at the end of 2018. The observed decrease may be an indicator of success for the new police initiatives. These initiatives added more approachable police officers to engage with the community, often on foot patrol and in highly frequented areas. They also improved illumination and monitoring of parking garages and other public places that are more prevalent Downtown.

## Results: Light rail stations

**Fig 10:**
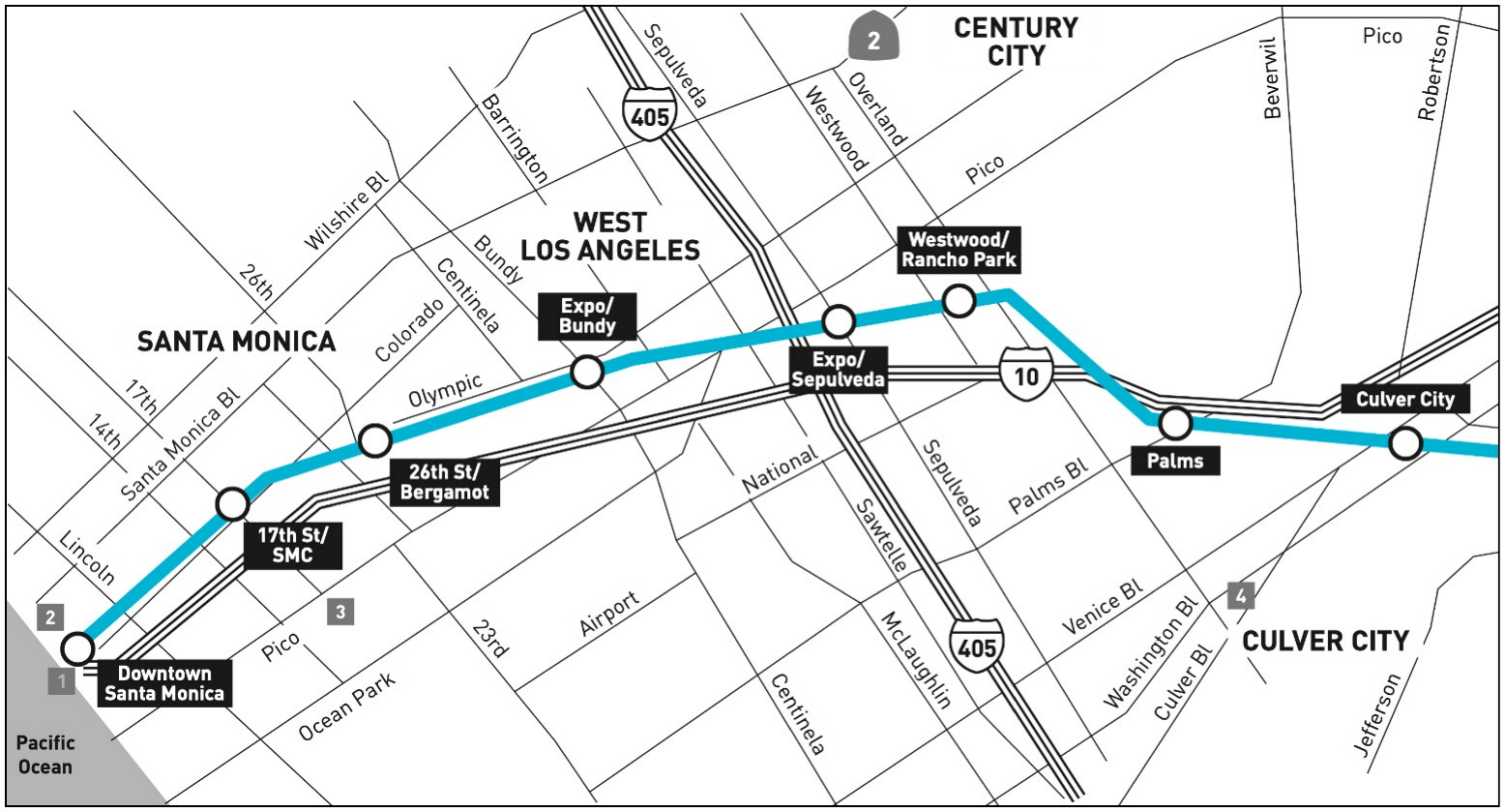
Map of the Metro Expo Line light rail extension to Santa Monica, CA. Operations began on May 20, 2016 at seven new stations, of which four are located within Santa Monica city borders. They are Expo/Bundy, 26^th^ Street/Bergamot, 17^th^ Street/Santa Monica College, and Downtown Santa Monica. The other three stations (Palms, Westwood/Rancho Park, Expo/Sepulveda) are within neighboring Culver City. Prior to May 20, 2016 the, Expo Line’s terminus was at the Culver City train stop. Picture courtesy of the Los Angeles Metro.

The Metro Expo Line extension was inaugurated on May 20, 2016, connecting Culver City to Santa Monica via light rail; see Fig 10. As discussed in Introduction, both the passage of Prop. 47 and the opening of the Expo Line have been blamed for increases in crime [26–28, 30–32].We aim to better understand whether and how the extension of the light rail affected reported criminal activity around the four new train stations located within Santa Monica municipal borders.

The possibility of mass transit leading to rising criminal activity, both inside stations surrounding them, is well-studied. While some results point to increases in crime [56–61], others suggest that mass transit does not necessarily lead to a decline in public safety [62–64]. Train and bus routes are usually concentrated in areas with high human activity, offering more opportunities for predatory crime. However, the impact of mass transit on crime is found to also depend on the overall demographic, socioeconomic, and land use contexts surrounding transit stops [62, 65, 66]. Finally, thoughtful architectural, lighting, and environmental design of the stations themselves may help reduce crime [62, 67, 68].

**Fig 11:**
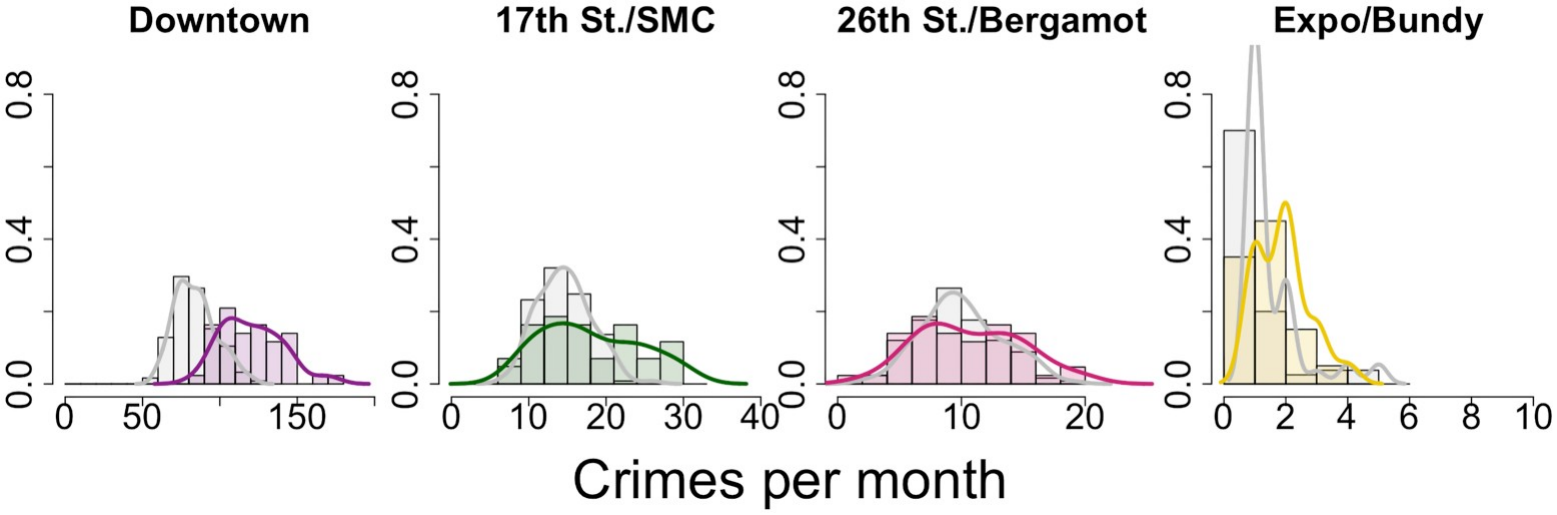
Reported crime near Expo Line light rail stations in Santa Monica, CA, 2006–2019, before and after opening of the light rail extension. These normalized histograms (bars) and densities (curves) provide monthly reported crime counts in a 450 meter radius around locations at which new stations opened in 2016. Gray represents the time period through May 2016, prior to this opening. Colored data is from the time period afterwards. Changes to the mean are statistically significant for the Downtown Santa Monica station, the 17^th^ Street/Santa Monica College station, and the Expo/Bundy station. They are not significant for the 26^th^ Street/Bergamot station. See Table 3.

To uncover possible associations between the opening of the Expo Line and reported crime, we focus on a radius of 450 meters centered around each of the four new Expo Line stops. We first neglect passage of Prop. 47 and consider the reported crime before and after inauguration of the light rail extension on May 20, 2016. For simplicity, we categorize the entire month of May 2016 as falling before opening of the Expo Line when creating monthly aggregates. See Fig 11 and Table 3 for results. The crime distributions shift to the right in a statistically significantly manner after opening of the Expo Line at the Downtown Santa Monica, the 17^th^ Street/Santa Monica College, and the Expo/Bundy stops. These shifts correspond to 43.1%, 23.4% and 25.6% increases in monthly crime rates, respectively. We observe no statistically significant changes around the 26^th^ Street/Bergamot station.

**Table 3:** Statistical tests on reported crime in the vicinity of light rail station locations in Santa Monica, CA, 2006–2019, before and after opening of the Expo Line light rail extension.

Train Station	before train {*μ*_b_, *σ*_b_}	after train {*μ*_a_, *σ*_a_}	*t*, *t*_*s*_	Significant?
Downtown	{84.26,13.56}	{120.53, 19.86}	11.12, 1.68	**yes, +43.1%**
17^th^ St./SMC	{14.65, 3.52}	{18.07, 6.38}	3.35, 1.68	**yes, +23.4%**
26^th^ St./Bergamot	{10.07, 3.30}	{10.72, 4.24}	0.91, 1.67	no
Expo/Bundy	{1.51, 0.99}	{1.90, 0.85}	1.76, 1.70	**yes, +25.6%**

We apply Welch’s t-test to the histogram data visualized in Fig 11. The last column indicates whether changes are statistically significant and shows percent changes to the mean. We highlight significant increases in boldface. The {*μ*_b_, *σ*_b_} quantities are monthly averages and standard deviations of reported crime before inauguration of the Expo Line calculated over *N*_b_ = 125 months. Their post-inauguration counterparts are {*μ*_a_, *σ*_a_} calculated over *N*_a_ = 43 months. The Welch’s t-test statistic, *t*, is compared to the Student’s t-distribution reference value *t*_s_.

**Fig 12:**
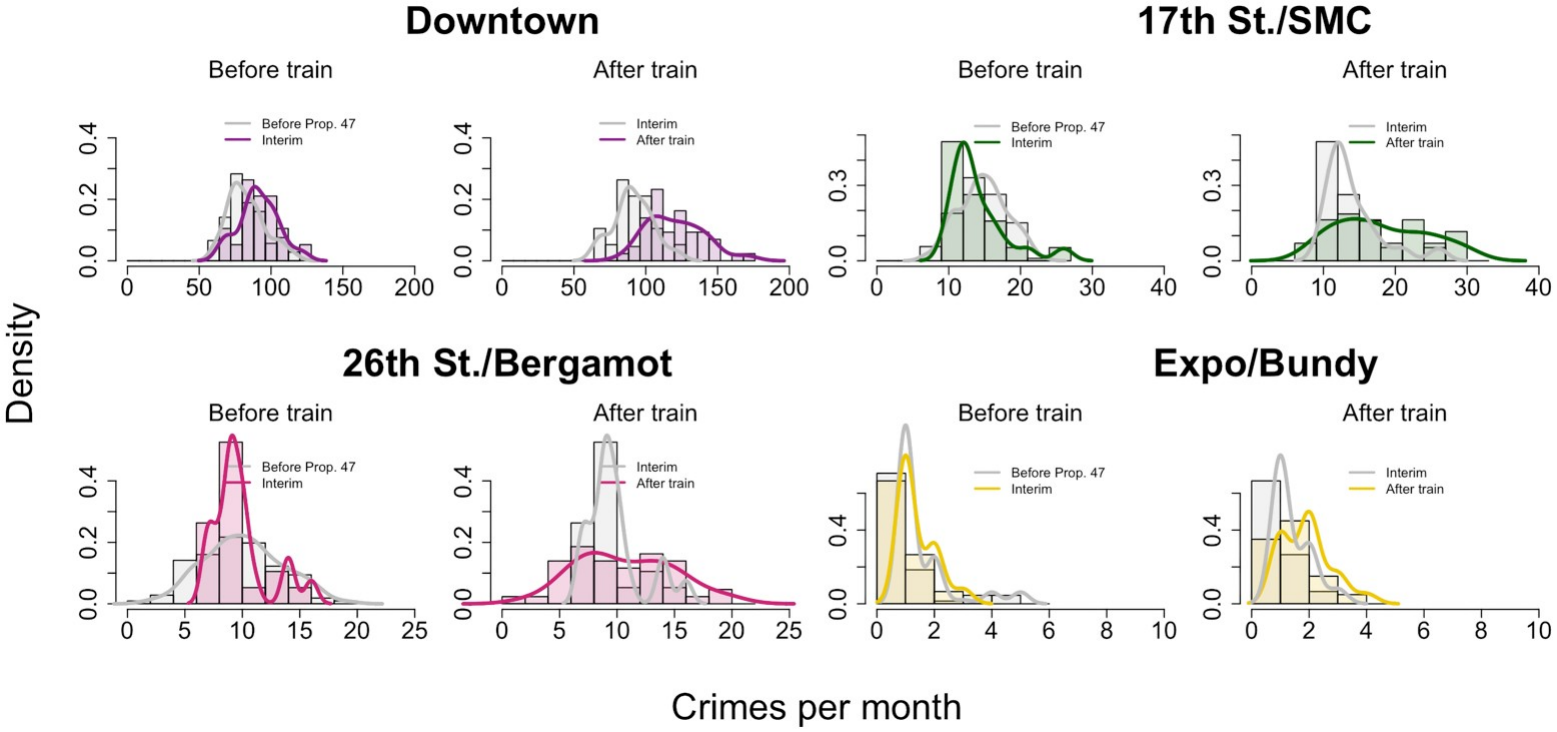
Reported crime near Expo Line light rail stations in Santa Monica, CA, during three successive time periods spanning 2006–2019. These normalized histograms (bars) and densities (curves) provide monthly reported crime counts in a 450 meter radius around locations at which new stations opened in 2016. The time periods are: (1) January, 2006—October, 2014, before implementation of Prop. 47; (2) November, 2014—May, 2016, between the implementation of Prop. 47 and the opening of the Expo Line; and (3) June, 2016—December, 2019, after the opening of the Expo Line. For each location, the left plot incorporates periods (1) and (2), and the right plot incorporates periods (2) and (3). Changes to the mean are not statistically significant prior to opening of the Expo Line (left plots) except for the Downtown station. After opening of the Expo Line (right plots), crimes increased significantly at the Downtown and 17^th^ Street/Santa Monica College stations. See Table 4.

**Fig 13:**
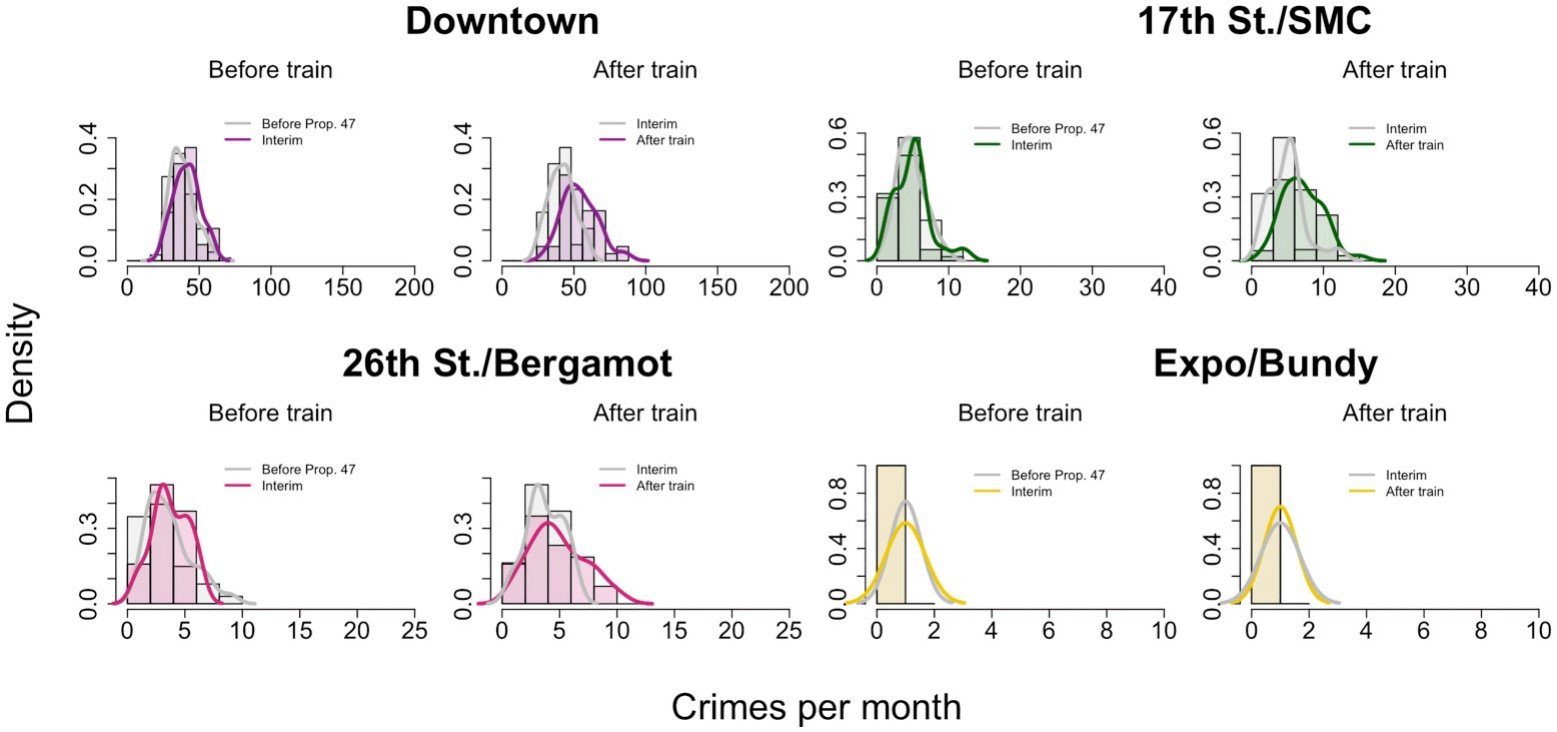
Reported Prop. 47 crime near Expo Line light rail stations in Santa Monica, CA, during three successive time periods spanning 2006–2019. This figure is analogous to Fig 12, but restricted to Prop. 47 crimes only. See Table 5 for statistical test results.

**Fig 14:**
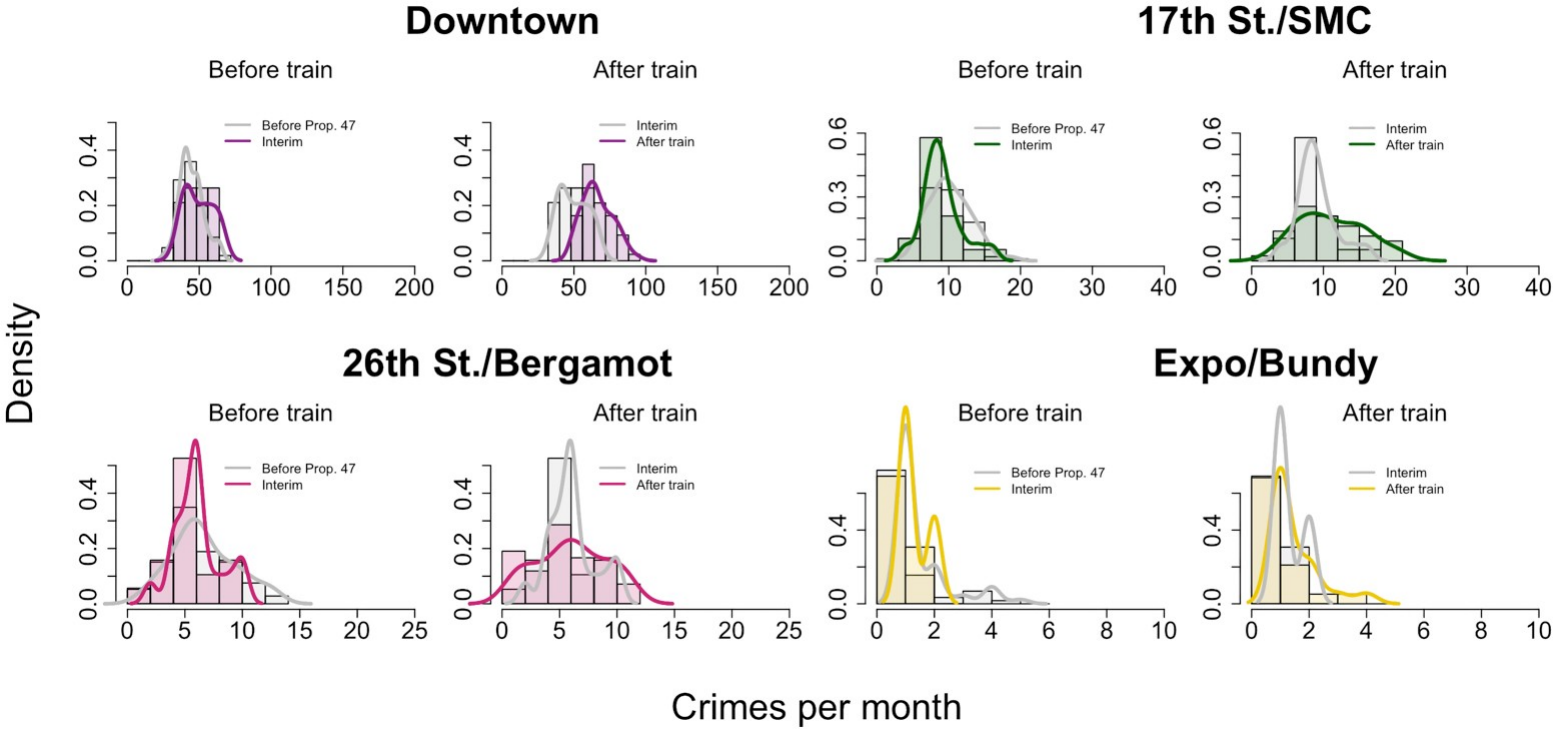
Reported non-Prop. 47 crime near Expo Line light rail stations in Santa Monica, CA, during three successive time periods spanning 2006–2019. This figure is analogous to Fig 12, but restricted to non-Prop. 47 crimes only. See Table 6 for statistical test results.

To separate the impacts of Prop. 47 from the opening of the Expo Line, we further refine our data by binning it into three time periods: (1) January, 2006—October, 2014, before implementation of Prop. 47; (2) November, 2014—May, 2016, between the implementation of Prop. 47 and the opening of the Expo Line; and (3) June, 2016—December, 2019, after the opening of the Expo Line. As a result of this stratification, we find at most one crime per month at the Expo/Bundy location. Given the paucity of data, we do not perform any further statistical analysis on this station. Results appear in Fig 12 and in Table 4. We find no significant increase in reported monthly crime near any of the remaining transit stops immediately after passage of Prop. 47 and prior to opening of the Expo Line (2014—2016). Reported crime instead increases dramatically after opening of the Expo Line at the Downtown and 17^th^ Street/Santa Monica College stations, by 32.2% and 28.6% respectively. Upon restricting our analysis to the reclassified crimes, as shown in Fig 13 and in Table 5, we find that reported Prop. 47 crimes increased significantly not only at the Downtown and 17^th^ Street/Santa Monica College stations, but also at 26^th^ Street/Bergamot station, by 30.6%, 38.6%, and 30.0% respectively. Finally, reported non-Prop. 47 crimes increased at the Downtown and 17^th^ Street/Santa Monica College stops by 33.5% and 23.1%, respectively, but did not significantly increase at 26^th^ Street/Bergamot; see Fig 14 and Table 6.

**Table 4:** Statistical tests on reported crime in the vicinity of light rail station locations in Santa Monica, CA, during three successive time periods spanning 2006–2019.

**All crimes; Monthly averages (2006-2014; 2014-2016)**				
**Train Station**	**before Prop. 47** *N*_b_ = 106 {*μ*_b_, *σ*_b_}	**after Prop. 47** *N*_a_ = 19 {*μ*_a_, *σ*_a_}	***t*, *t*_*s*_**	**Significant?**
Downtown	{83.01, 13.14}	{91.21, 13.41}	2.46, 1.71	yes, +9.9%
17^th^ St./SMC	{14.75, 3.41}	{14.05, 3.91}	0.73, 1.71	no
26^th^ St./Bergamot	{10.12, 3.42}	{9.79, 2.40}	0.52, 1.70	no
Expo/Bundy	{0.94, 0.95}	{1.11, 0.60}	0.97, 1.70	N/A
**All crimes; Monthly averages (2014-2016; 2016-2019)**				
**Train Station**	**before train** *N*_b_ = 19 {*μ*_b_, *σ*_b_}	**after train** *N*_a_ = 43 {*μ*_a_, *σ*_a_}	***t*, *t*_*s*_**	**Significant?**
Downtown	{91.21, 13.41}	{120.53, 19.62}	6.83, 1.68	yes, +32.2%
17^th^ St./SMC	{14.05, 3.91}	{18.07, 6.30}	3.05, 1.68	yes, +28.6%
26^th^ St./Bergamot	{9.79, 2.40}	{10.72, 4.19}	1.11, 1.68	no
Expo/Bundy	{1.11, 0.60}	{0.88,0.90}	0.97, 1.68	N/A

The time periods are: (1) January, 2006—October, 2014, before implementation of Prop. 47; (2) November, 2014—May, 2016, between the implementation of Prop. 47 and the opening of the Expo Line; and (3) June, 2016—December, 2019, after the opening of the Expo Line. We apply Welch’s t-test to the histogram data visualized in Fig 12. The last column indicates whether changes are statistically significant and shows percent changes to the mean. The top table compares time periods (1) and (2), and the bottom table compares (2) and (3). The {*μ*_b_, *σ*_b_} quantities are monthly averages and standard deviations of crime before the end of a time period, calculated over *N*_b_ months. Their counter parts for afterwards are {*μ*_a_, *σ*_a_}, calculated over *N*_a_ months. The Welch’s t-test statistic, *t*, is compared to the Student’s t-distribution reference value *t*_s_. There is not sufficient data for meaningful conclusions at Expo/Bundy.

**Table 5:** Statistical tests on reported Prop. 47 crime in the vicinity of light rail station locations in Santa Monica, CA, during three successive time periods spanning 2006–2019.

**Prop. 47 crimes; Monthly averages (2006-2014; 2014-2016)**				
**Train station**	**before Prop. 47** *N*_b_ = 106 {*μ*_b_, *σ*_b_}	**after Prop. 47** *N*_a_ = 19 {*μ*_a_, *σ*_a_}	***t*, *t*_*s*_**	**Significant?**
Downtown	{38.38, 8.93}	{41.47, 8.95}	1.39, 1.71	no
17^th^ St./SMC	{4.74, 2.00}	{5.00, 2.52}	0.44, 1.72	no
26^th^ St./Bergamot	{3.48, 1.90}	{3.74, 1.56}	0.65, 1.70	no
Expo/Bundy	{0.12, 0.31}	{0.21, 0.36}	1.00, 1.72	N/A
**Prop. 47 crimes; Monthly averages (2014-2016; 2016-2019)**				
**Train station**	**before train** *N*_b_ = 19 {*μ*_b_, *σ*_b_}	**after train** *N*_a_ = 43 {*μ*_a_, *σ*_a_}	***t*, *t*_*s*_**	**Significant?**
Downtown	{41.47, 8.95}	{54.14, 12.23}	4.57, 1.71	yes, +30.6%
17^th^ St./SMC	{5.00, 2.52}	{6.93, 2.74}	2.76, 1.70	yes, +38.6%
26^th^ St./Bergamot	{3.74, 1.56}	{4.86, 2.41}	2.19, 1.68	yes, +30.0%
Expo/Bundy	{0.21, 0.36}	{0.23, 0.37}	0.22, 1.70	N/A

We apply Welch’s t-test to the histogram data visualized in Fig 13. This table is analogous to Table 4, but restricted to Prop. 47 crimes only.

**Table 6:** Statistical tests on reported non-Prop. 47 crime in the vicinity of light rail station locations in Santa Monica, CA, during three successive time periods spanning 2006–2019.

**non-Prop. 47 crimes; Monthly averages (2006-2014; 2014-2016)**				
**Train Station**	**before** {*μ*_b_, *σ*_b_, *N*_b_ = 106}	**after** {*μ*_a_, *σ*_a_, *N*_a_ = 19}	***t*, *t*_*s*_**	**Significant?**
Downtown	{44.63, 8.38}	{49.74, 9.80}	2.14, 1.72	yes, +11.44%
17^th^ St./SMC	{10.02, 2.93}	{9.05, 2.70 }	1.42, 1.71	no
26^th^ St./Bergamot	{6.08, 2.65}	{5.42, 2.23}	1.16, 1.70	no
Expo/Bundy	{0.76, 0.85}	{0.89, 0.51}	0.91, 1.70	N/A
**non-Prop. 47 crimes; Monthly averages (2014-2016; 2016-2019)**				
**Train Station**	**before train** {*μ*_b_, *σ*_b_, *N*_b_ = 19}	**after train** {*μ*_a_, *σ*_a_, *N*_a_ = 43}	***t*, *t*_*s*_**	**Significant?**
Downtown	{49.74, 9.80}	{66.40, 10.62}	6.01, 1.70	yes, +33.5%
17^th^ St./SMC	{9.05, 2.70}	{11.14, 4.61}	2.23, 1.68	yes, +23.1%
26^th^ St./Bergamot	{5.42, 2.23}	{4.77, 2.69}	1.00, 1.68	no
Expo/Bundy	{0.89, 0.51}	{0.53, 0.60}	2.42, 1.68	N/A

We apply Welch’s t-test to the histogram data visualized in Fig 14. This table is analogous to Table 4, but restricted to non-Prop. 47 crimes only.

## Discussion and conclusions

Using a publicly available database compiled and maintained by the Santa Monica Police Department, we investigated whether passage of Proposition 47 in the state of California was associated with any change in reported crime. We specifically focused on crimes that were directly affected by legislative change and were reclassified from felonies to misdemeanors. Our analysis shows that overall the monthly reports of these crimes (larceny, fraud, possession of narcotics, forgery, receiving/possessing stolen property) increased by about 15% after implementation of Prop. 47 in November, 2014. By contrast, reported non-reclassified crime decreased by approximately 9% after the new legislation became effective. We used a Welch’s t-test to verify that the reclassified crime distribution shift from less crime before November, 2014 to more crime afterwards was statistically significant. Additionally, we used a signal decomposition to isolate the main reported crime trends from seasonal effects. Using change-point analysis, we identified a discontinuity in reported crime trend at the end of 2014. A segmented regression analysis on the monthly time series led us to identify November, 2014 as the main breakpoint. We also identified a secondary discontinuity in the crime trend and a secondary break-point in the monthly time series, both occurring towards the end of 2018, indicating a decrease in reported crime concurrent with new policing efforts. While these changes to policing are too recent to reverse the overall crime surge observed after the 2014 implementation of Prop. 47, and while it is unclear what the longer term implications of the new initiatives will be, our results perhaps suggest that community partnerships, a responsive and outward facing police force, and targeted measures may be associated with a reduction in reported crime.

We also considered the impact of Prop. 47 on the eight neighborhoods that comprise the city and verified that the largest monthly change in crime, at +37.2%, occurred Downtown, an area with many opportunistic crime attractors including nightlife, tourists, dining venues, and shopping centers. Finally, we examined the effects of the opening of the Expo Line on monthly crime rates within 450 meters of the locations of four new transit stations. We found that reported crime increased significantly at the Downtown Santa Monica station, the 17^th^ Street/Santa Monica College station, and the Expo/Bundy station. Prop. 47 and non-Prop. 47 percent increases were comparable at the Downtown Santa Monica stop, at +30.6% and +33.5% respectively. In contrast, at the 17^th^ Street/Santa Monica College stop, the increase for reported Prop. 47 crimes was +38.6%, much higher than for non-Prop. 47 ones, at +23.1%.

Several observations are in order. The city of Santa Monica does not report the monetary value of relevant crimes. Partitioning of the data into reclassified vs. non-reclassified offenses is thus based on our best estimate of which crimes would, on average, fall under the 950 USD threshold, one of the conditions specified by Prop. 47 for reclassification. For example, the initiative applies to grand theft auto but only for vehicles worth less than 950 USD. Since the typical value of stolen cars in Santa Monica surpasses this threshold, we do not include grand theft auto in the list of Prop. 47 crimes. It is clear that exceptions may exist and that our partitioning may have introduced errors; however, due to the large data sample, we expect these not to be systematic and not to have significantly affected our results. Other biases could arise from unreported victimizations differentially affecting the reclassified vs. non-reclassified crime categories. For instance, the US Department of Justice estimates that the average annual incidence of unreported larceny was 41% nationwide over the 2006—2010 period; for motor vehicle theft the same figure was 17% [69]. Similarly, reporting rates could change over time in response to changing perceptions of the effectiveness of reporting crimes. Finally, the period between passage of Prop. 47 in November, 2014 and the opening of the Expo Line in May 2016 is only 18 months. Compounding effects of the two events may have led to the observed increases in crime; disentangling their overlap may require more discriminants than the data analyzed here.

Possible extensions of this work could involve analyzing reported crime in neighboring cities that share similar socio-economic backgrounds with Santa Monica, such as Culver City (population 39,000), Pasadena (population 138,000) and Glendale (population 203,000). These cities are all within Los Angeles County, although they are less touristic, and are not directly adjacent to the Pacific Ocean. Similarly, it could be elucidating to study reported crime near the other three new Expo Line stations operating in Culver City (Palms, Westwood/Rancho Park, Expo/Sepulveda) to compare and contrast results between the two municipalities. A longer term monitoring of crime in Santa Monica is also desirable. This would allow us to determine whether the decrease in the number of reported monthly crimes observed in late 2018 persists or stabilizes over time. It would also allow us to better discern the effects of the Expo Line, since reported crime may temporarily increase around newly opened stations and settle back to original levels once novelty effects subside [59]. Unfortunately, such studies may not be possible due to the COVID-19 pandemic that severely impacted the global economy. Suspension of all non-essential activities and stay-at-home orders within the city of Santa Monica, as well as reduction to service and ridership of the Expo Line, do not allow for a meaningful, continuous, long-term analysis. Although conducted over short time frames, both after passage of Prop. 47 and the inauguration of the Expo Line, our results do suggest that neighborhood characteristics may influence reported crime, since areas with more opportunities for crime appear are associated with the light rail extension and the new law. Similarly, our results hint that community-based policing and targeted interventions may help reduce (reported) crime.

Finally, although we find a rise in reported reclassified crimes that coincides with passage of Prop. 47, our research does not provide a definite causative explanation for it. While it is possible that the new law directly motivated offenders to commit more reclassified crimes, there may also be other relevant explanations. Among them are the increased attention of police, heightened public awareness, and more reporting, all of which may have been influenced by media coverage. The observed rise may also be a loose manifestation of the well known Hawthorne effect, whereby individuals modify their behavior as a result of being part of an experiment or study [70, 71]. In this case, crime reporting behavior could have been affected by awareness of the changes brought by Prop. 47. Similarly, the decrease in the number of reported reclassified crimes observed in late 2018 may be due to shifts in policing, but also due to fading of Prop. 47 awareness, or habituation. We hope that these and other considerations relevant to public utility, respect for human rights, and existence of socioeconomic disparities, will be used in combination with our results to assess the overall effect of Prop. 47.

## Supporting information

S1 Appendix STL decomposition and change-point analysis. We present technical details related to STL decomposition and change-point analysis, as well as supplementary results obtained by applying these methods to reported crime data for the eight neighborhoods of Santa Monica, CA, 2006–2019.
(PDF)
