# Correction: Impacts of California Proposition 47 on crime in Santa Monica, California

**DOI:** 10.1371/journal.pone.0314701

**Published:** 2024-11-25

**Authors:** Jennifer Crodelle, Celeste Vallejo, Markus Schmidtchen, Chad M. Topaz, Maria R. D’Orsogna

The color scheme of eight neighborhoods in Fig 8 is incorrect. Please see the correct Fig 8 here.

**Fig 8:**
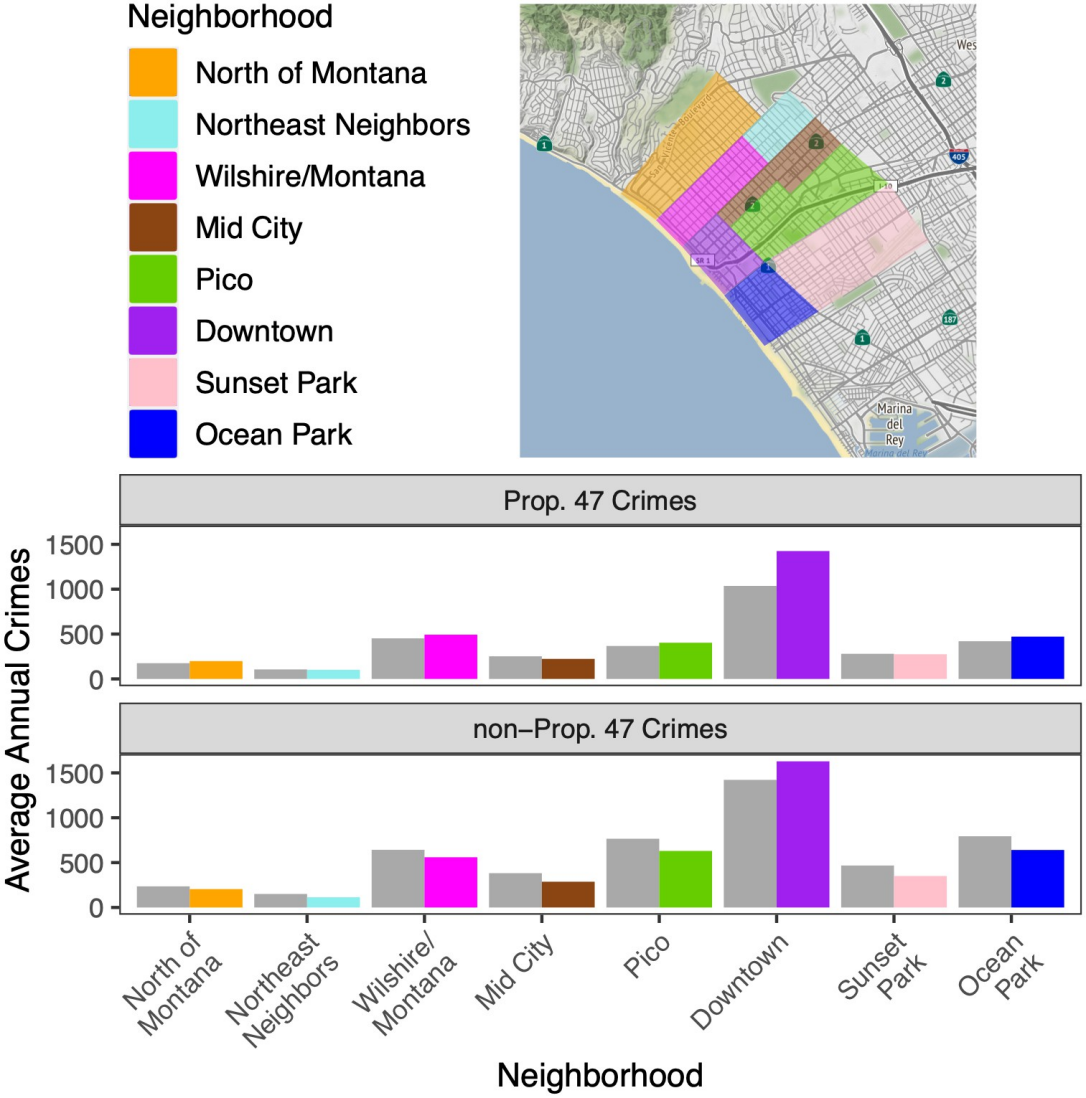
Neighborhoods of Santa Monica, CA, and their average annual reported crimes. (Top) The eight neighborhoods of the city of Santa Monica. To the north of Santa Monica is Pacific Palisades, to the south is Venice, and to the east is West Los Angeles, which are all part of the city of Los Angeles proper. To the west is the Pacific Ocean. Created using OpenStreetMaps. (Bottom): Average annual reported crimes in each neighborhood for Prop. 47 crimes (top) and non-Prop. 47 crimes (bottom). Gray bars indicate the average prior to implementation of the new law in November, 2014. The color-coded bars represent the averages after Prop. 47 came into effect.
