# Supplementary material for: Impacts of California Proposition 47 on crime in Santa Monica, California
Source: PLoS One. 2021 May 19;16(5):e0251199. doi: 10.1371/journal.pone.0251199 (PMC8133468; doi:10.1371/journal.pone.0251199)
Supplement: S1 Appendix — We present technical details related to STL decomposition and change-point analysis, as well as supplementary results obtained by applying these methods to reported crime data for the eight neighborhoods of Santa Monica, CA, 2006–2019. (PDF) [file pone.0251199.s001.pdf]

S1 Appendix

Jennifer Crodelle[1⋆], Celeste Vallejo[2⋆], Markus Schmidtchen[3], Chad M. Topaz[4,5], and Maria R. D'Orsogna[6,7‡]

1 Department of Mathematics, Middlebury College, Middlebury, VT, USA
2 Mathematical Biosciences Institute, The Ohio State University, Columbus, OH, USA
3 Laboratoire Jacques-Louis Lions, Sorbonne Université, Paris, France
4 Institute for the Quantitative Study of Inclusion, Diversity, and Equity, Williamstown, MA, USA
5 Department of Mathematics and Statistics, Williams College, Williamstown, MA, USA
6 Department of Computational Medicine, UCLA, Los Angeles, CA, USA
7 Department of Mathematics, CSUN, Los Angeles, CA, USA

⋆ These authors contributed equally to this work.
‡dorsogna@csun.edu

This appendix contains additional details of our Seasonal and Trend decomposition using Loess (STL) and change-point analyses.

Seasonal and Trend decomposition using Loess (STL)

We perform STL decomposition via the 'stl' function in the R software environment [1]. The STL algorithm is an iterative process that uses the monthly time series $Y(t)$ as input data as well as user-specified initial trend and seasonality estimates. The latter are typically null sets. In each iteration the data is cleared of the current trend estimate and broken into cycle-subseries, one for each of the data points within a period. In our case, since we have monthly data with a periodicity of $n_{\mathrm{p}} = 12$ months, twelve cycle-subseries arise, one for each month.

The algorithm obtains a new seasonal series through a combination of loess (locally estimated scatterplot smoothing) polynomial regression with given weights, and moving averages performed on each of the cycle-subseries. The loess regression ensures that the obtained seasonality is defined for all times, not just when data points are available; the moving average procedures guarantee the mean is nearly zero. The input data is then cleared of the newly derived seasonal effect and a temporary trend is obtained, once more using a loess polynomial regression. The freshly derived trend and seasonal estimates are then used as inputs for the next iteration. Any residual elements are included in the remainder and used to compute robustness weights, to reduce the influence of transient, aberrant behavior in the data on the trend and seasonal components. The procedure is run until a pre-set convergence is reached; typically two loops suffice.

Two window lengths must be specified to apply the loess regression in the 'stl' function: w_{season} (the s.window argument in R) and w_{trend} (the t.window argument in R). Since we do not assume seasonal patterns to have significantly evolved over the thirteen year time span under investigation, we use the entire data to perform the loess seasonal smoothing analysis and set s.window = periodic. Effectively, the monthly cycle-subseries are smoothed using weighted averages over all the relevant monthly data.

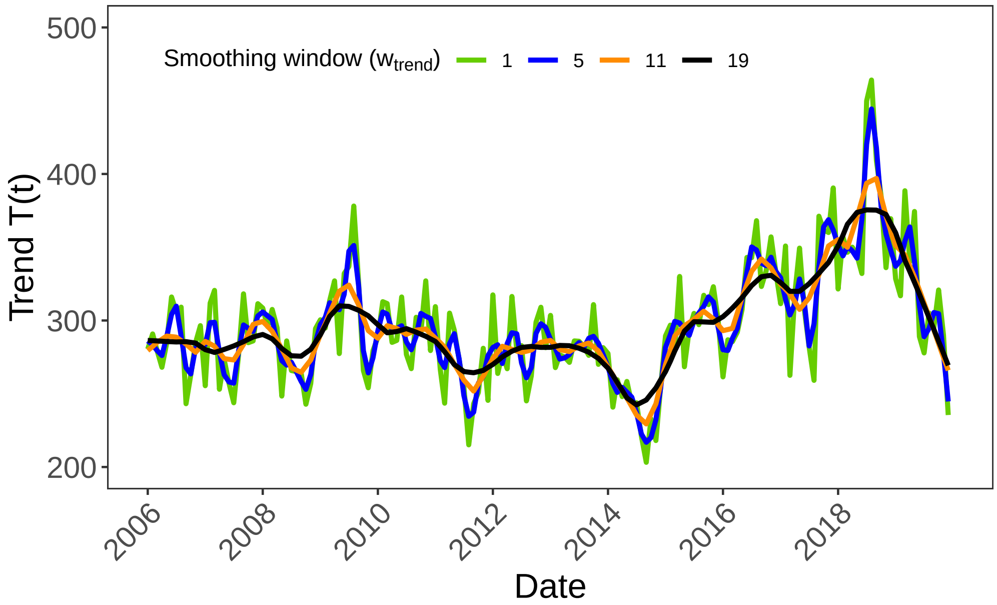

Figure S1. Trends $T(t)$ evaluated on Prop. 47 crimes using decomposition using loess from the R statistical package with varying window lengths w_{trend}. As w_{trend} increases, the corresponding $T(t)$ becomes smoother. Unless otherwise stated, we set $w_{\text{trend}} = 19$ months (black curve) as determined by Eq. (1).

If seasonality were expected to change over the 2006 − 2019 arc, the analysis would have to be performed using a more restricted window, so that older seasonal patterns would not affect more recent ones. Finally, w_{trend} is assumed to be an odd integer and is set following standard procedures [1] as

$$\text{t.window} = \text{Nextodd}\left[\text{Ceiling}\left(\frac{1.5\,\text{n}_\text{p}}{1 - 1.5\,/\text{s.window}}\right)\right]. \tag{1}$$

Here, NextOdd(.) is the smallest odd integer greater than, or equal to its argument, and Ceiling(.) is the smallest integer greater than or equal to its argument. Eq. (1) yields values for w_{trend} that are known to prevent overlaps between the trend and seasonal components.

The value of w_{trend} plays a fundamental role in the decomposition process: as this parameter increases more points are used in the smoothing process, and sharper trends may be identified. Increases to w_{trend} however may also minimize, eliminate, or shift peaks and valleys. Our data set leads to $w_{\text{trend}} = 19$ months. Unless otherwise noted, we use this value as the default; when more resolution is necessary, for example to investigate trends around November 2014, smaller window lengths are used. Fig. S1 shows various smoothed trend curves for different w_{trend}: note how the minimum located towards the end of 2014 shifts in time and depth as w_{trend} is modified.

Change-point analysis

Our change-point analyses are performed using the R package 'mosum' [2] which uses a moving sum to average over subsets of the data; the size of the subset G is termed bandwidth. For a given data point, the prior and after averages over the bandwidth are evaluated together with the respective variances; the first and last G points are discarded. A mosum statistic is then constructed as the difference between the after and

prior averages divided by an ad-hoc standard deviation, which may be chosen as the root of the average, minimum, or maximum of the after and prior variances. We select the average. The mosum statistic is then compared with a threshold derived from an asymptotic distribution that depends on the bandwidth, the size of the entire time series, and a significance level chosen by the user. If the mosum statistic exceeds this critical threshold then the null hypothesis, of no change-points, is rejected in favor of the alternative one; the corresponding data point is now considered a change-point estimator.

Other criteria must be met in order to identify true change-points from the estimators above. These criteria are imposed so that spurious peaks are discarded and multiple estimates pertaining to the same underlying true change-point are avoided. The η-criterion is used to set the minimum distance between possible change-points at ηG, whereas the ϵ-criterion imposes that not only the putative change-point but an entire neighborhood of minimum width ϵG, with $\epsilon < 1$, centered about it must surpass the threshold. Confidence intervals for the change-point locations are evaluated using bootstrap methods as illustrated in [2].

STL decomposition in the eight Santa Monica neighborhoods

We plot here the monthly crime time series $Y(t)$ for each of the eight neighborhoods in the city of Santa Monica for both Prop. 47 and non-Prop. 47 crimes. We also present the respective trend $T(t)$, seasonality $S(t)$ and remainder $R(t)$ components through an STL decomposition. The greatest trend increase for Prop. 47 crimes is observed in Downtown, together with a sharp decrease starting in late 2018.

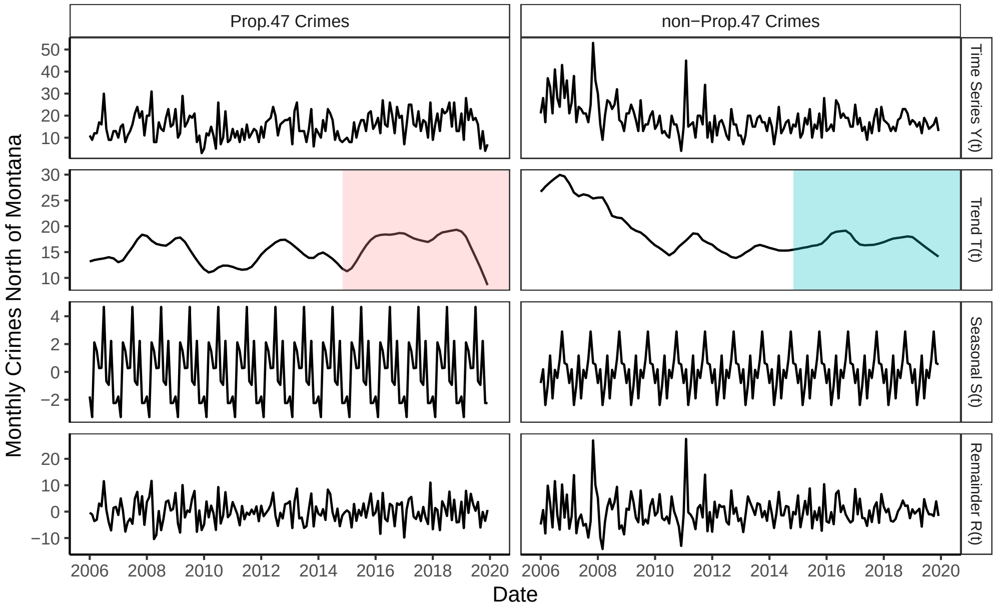

Figure S2. STL decomposition of monthly reported crime in the North of Montana neighborhood of Santa Monica, CA, 2006 − 2019.

References

1. R Core Team. R: A language and environment for statistical computing; 2018. Available from: https://www.R-project.org/.

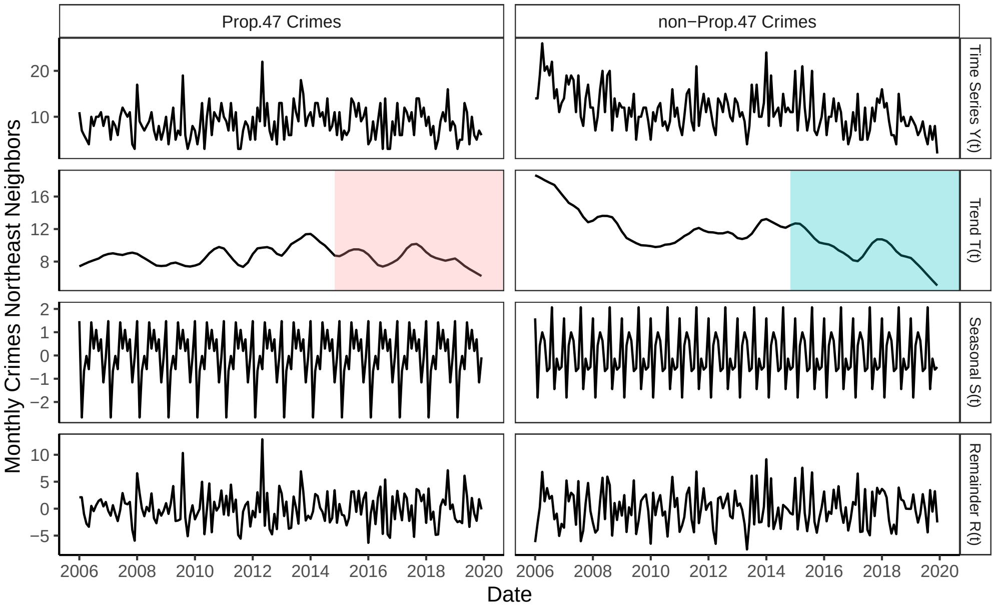

Figure S3. STL decomposition of monthly reported crime in the Wilshire/Montana neighborhood of Santa Monica, CA, 2006 – 2019.

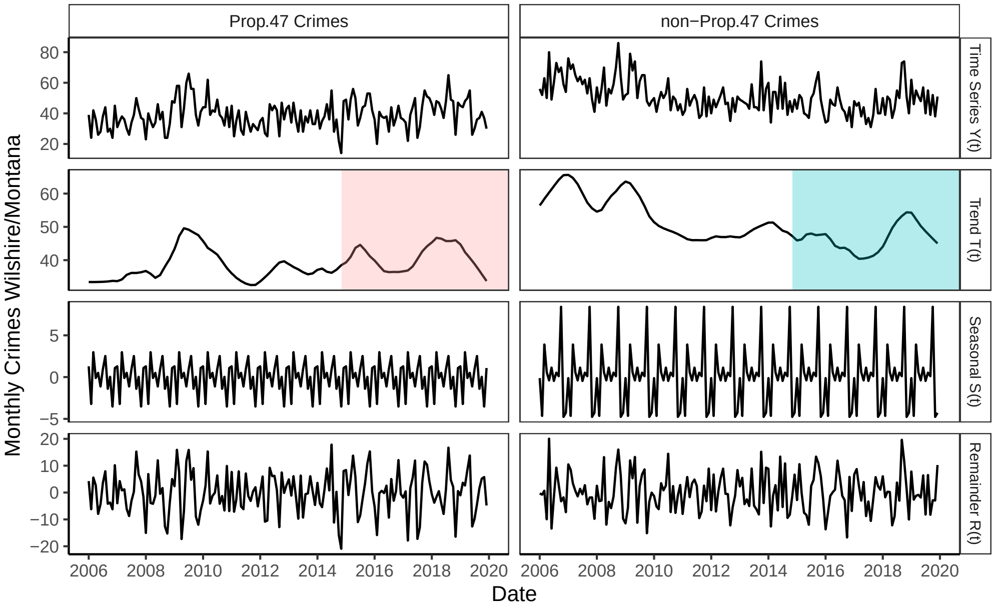

Figure S4. STL decomposition of monthly reported crime in the Northeast Neighbors neighborhood of Santa Monica, CA, 2006 – 2019.

2. Meier A, Cho H, Kirch C. Mosum: Moving sum based procedures for changes in the mean; 2019. Available from: https://CRAN.R-project.org/package=mosum.

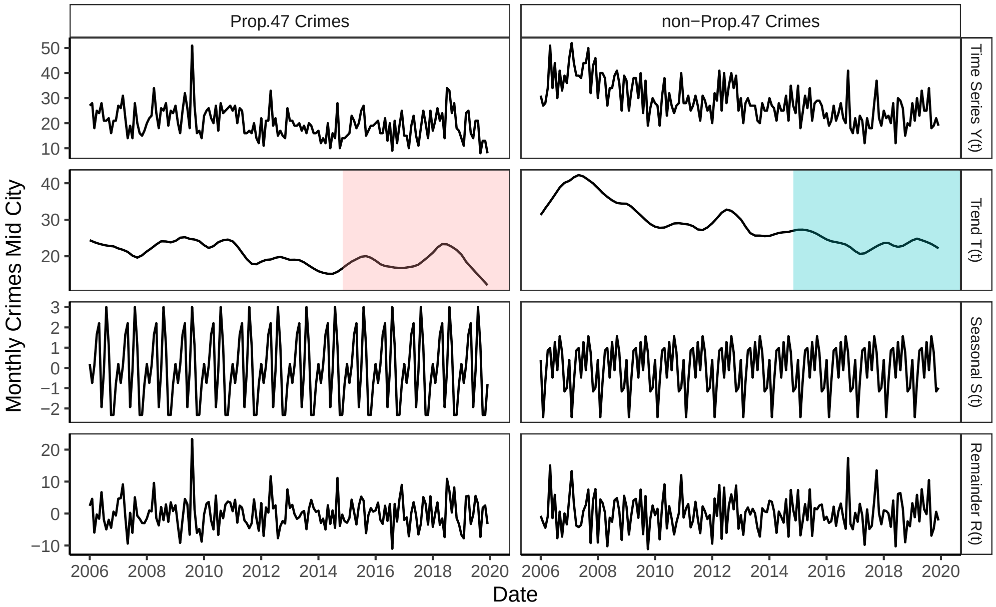

Figure S5. STL decomposition of monthly reported crime in the Mid City neighborhood of Santa Monica, CA, 2006 – 2019.

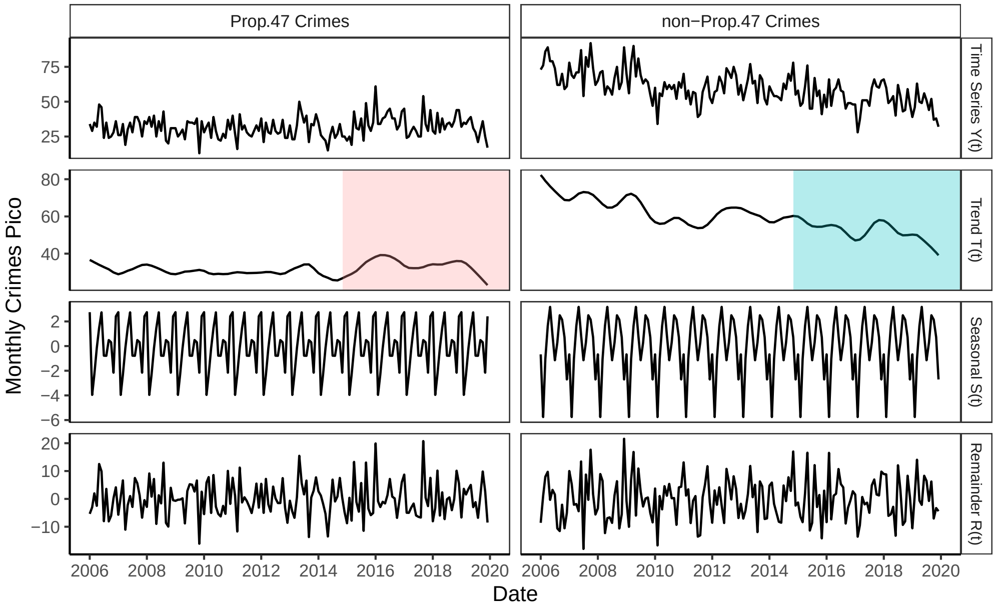

Figure S6. STL decomposition of monthly reported crime in the Downtown neighborhood of Santa Monica, CA, 2006 – 2019.

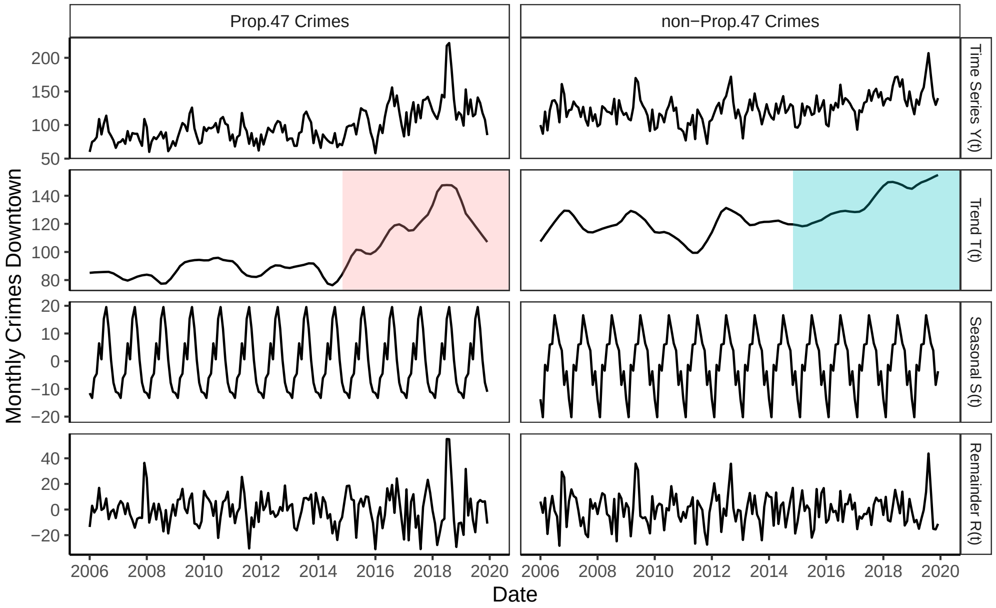

Figure S7. STL decomposition of monthly reported crime in the Pico neighborhood of Santa Monica, CA, 2006 – 2019.

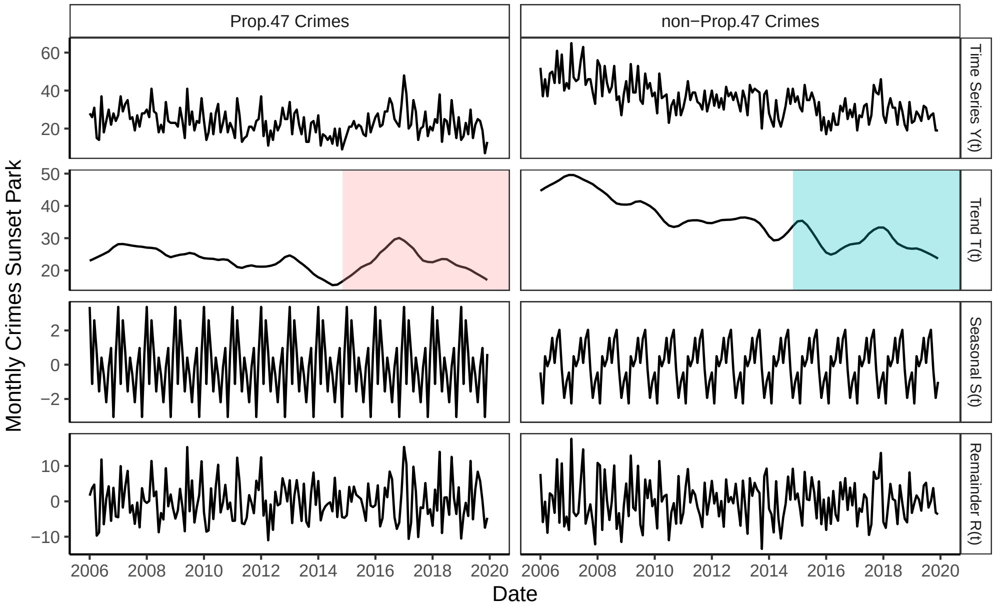

Figure S8. STL decomposition of monthly reported crime in the Sunset Park neighborhood of Santa Monica, CA, 2006 – 2019.

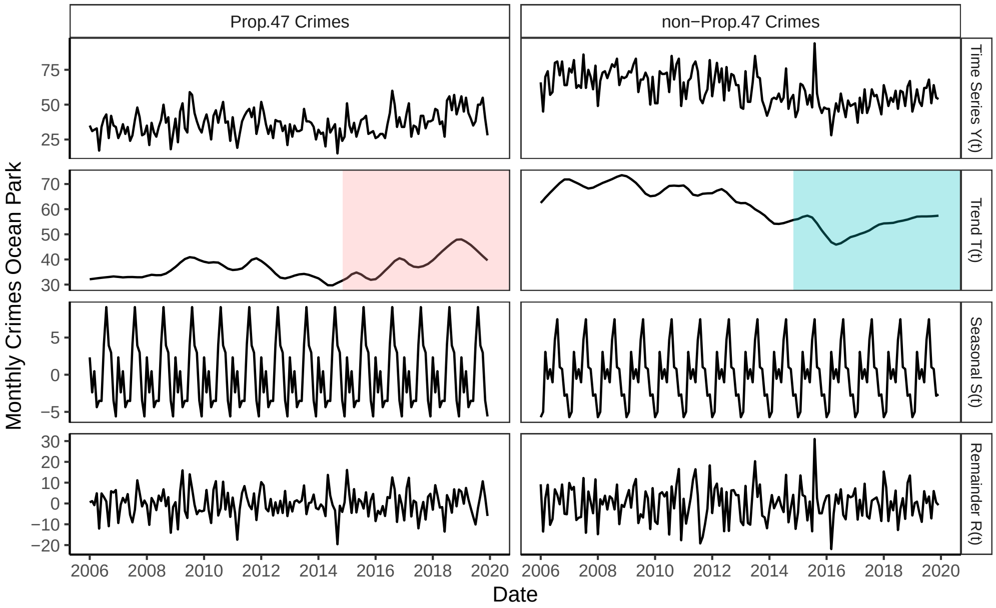

Figure S9. STL decomposition of monthly reported crime in the Ocean Park neighborhood of Santa Monica, CA, 2006 – 2019.
